# Australian Cool-Season Pulse Seed-Borne Virus Research: 3 Pea Seed-Borne Mosaic Virus

**DOI:** 10.3390/v18030322

**Published:** 2026-03-04

**Authors:** Roger A. C. Jones, Benjamin S. Congdon

**Affiliations:** 1 UWA Institute of Agriculture, University of Western Australia, Crawley, WA 6009, Australia; 2 Department of Primary Industries and Regional Development, Jandacott, Perth, WA 6164, Australia; benjamin.congdon@dpird.wa.gov.au

**Keywords:** history, grain legumes, virus diseases, seed-transmitted viruses, *Potyvirus pisumsemenportati*, epidemiology, management, losses, achievements, future research priorities

## Abstract

Here, we adopt an historical approach towards reviewing research since the 1970s on the seed-borne virus diseases of cool-season pulses caused by pea seed-borne mosaic virus (PSbMV) in Australia’s grain cropping regions. All relevant investigations concerning the principal cool-season pulse crops infected; field pea, lentil, faba bean, chickpea, and the minor ones, *Lathyrus* species, vetches and narbon bean, are covered. However, as the PSbMV field pea pathosystem is the most studied, this receives greatest emphasis. The review starts with brief background information, and by describing the disease symptoms caused and the advances in sample testing procedures. Next, findings from past PSbMV studies are covered in greater detail including transmission by aphids, contact and seeds; occurrence in crops and seed stocks; pathotypes and genetic diversity; host resistance; and phytosanitary, cultural and chemical control measures. What these studies found about PSbMV biology, epidemiology and control is emphasized by describing past glasshouse and field experimentation. Then, practical research outcomes identifying PSbMV’s epidemic drivers, forecasting its epidemics and devising an integrated disease management strategy are emphasized. Examples of images that illustrate past investigations and research outputs are provided. Finally, principal research achievements and priorities for future Australian PSbMV cool-season pulse research are highlighted.

## 1. Introduction

Here, we contribute the last of three historical reviews of Australian research on major seed-borne virus diseases of cool-season pulse crops. Volume 1 of this series provided background information about Australia’s large cool-season pulse industry, general plant virus epidemiology and management principles, future threats to achieving successful virus disease control and general recommendations for future research [1]. However, its main focus was on providing a detailed historical description from the 1940s up to the present time of the substantial amount of Australian research on the seed-borne virus diseases caused by cucumber mosaic virus (CMV; genus *Cucumovirus*, family *Bromoviridae*) and alfalfa mosaic virus (AMV; genus *Alfamovirus,* family *Bromoviridae*), and how to manage them effectively. In addition, it described past Australian studies with five less important seed-borne viruses sometimes found infecting cool-season pulse crops in Australia, broad bean stain virus (genus *Comovirus*, family *Secoviridae*), broad bean true mosaic virus (genus *Comovirus*, family *Secoviridae*), broad bean wilt virus (genus *Fabavirus*, family *Secoviridae*), cowpea mild mottle virus (genus *Carlavirus*, family *Betaflexiviridae*) and peanut mottle virus (genus *Potyvirus*, family *Potyviridae*). Volume 2 provided a historical account from the 1940s to the present of the extensive Australian research on the diseases bean yellow mosaic virus (BYMV; genus *Potyvirus*, family *Potyviridae*) causes in cool-season pulses and how to control them successfully [2]. In addition to describing past studies revealing information on pathosystem biology, both Volumes placed special emphasis on describing: (i) field and glasshouse experiments on phytosanitary, cultural and host resistance measures that enabled effective integrated disease management (IDM) strategies to be devised; and (ii) field experiments studying the spatial and temporal dynamics of virus spread that provided new knowledge of factors driving epidemics, which was needed for the development of effective forecasting models and decision support systems. Examples from an extensive collection of color images derived from field-based research from the last 40 years was included within a series of Figures to help the reader comprehend the scope of the research described and what it revealed. With CMV and BYMV, by far the greatest proportion of Australian research concerns the diseases they cause in lupin (*Lupinus*) crop species, especially narrow-leafed lupin (*L. angustifolius*) and white lupin (*L. albus*), so past research findings with it are covered in considerable detail. For CMV, lentil (*Lens culinaris*) was identified as the crop in greatest need of future research activity. With BYMV in pulse crops apart from lupin, most past research has focused on faba bean (*Vicia faba*), which constitutes the crop warranting greatest additional research apart from lupin. Less research has been done with AMV with which lentil was identified as the pulse crop in greatest need of future research activity. The most important achievements that resulted from eight decades of Australian research on seed-borne virus diseases of cool-season pulses caused by CMV, AMV and BYMV, and recommendations regarding future research priorities for each of them, were highlighted [1,2].

This volume, No. 3, covers the considerable amount of Australian research undertaken since the late 1970s on the seed-borne diseases that pea seed-borne mosaic virus (PSbMV; genus *Potyvirus*, family *Potyviridae*) infection elicits in cool-season pulse crops and their management. All major cool-season pulses grown in Australia apart from lupin become infected with PSbMV, as do all the minor cool-season pulses also grown on the continent. Amongst these pulses, the disease PSbMV causes in field pea (*Pisum sativum*) is the most economically important and extensively studied [3,4,5,6,7,8,9,10]. Therefore, the information reviewed mostly comes from studies involving the PSbMV/field pea pathosystem. However, all available past findings arising from Australian PSbMV studies with other cool-season pulses, most of which concern faba bean, lentil and chickpea, are also covered. The same approach as that used in Volume 2 is adopted, including use of color images to illustrate past investigations and research outputs. However, more emphasis is placed on biological studies in the glasshouse because fewer field experiments on cultural or chemical control measures, and none studying the spatial and temporal dynamics of spread, were undertaken. Finally, practical research outcomes, important research achievements and future research needs are emphasized.

## 2. Background Information

PSbMV was first described in 1966 in Czechoslovakia [11] and in 1967 in Japan [12]. It infects at least 47 species in 12 dicotyledonous families, causing serious diseases in some legume species but mostly asymptomatic infection in non-legume hosts [13,14]. It is transmitted non-persistently by many aphid species, which include *Acyrthosiphon kondoi*, *A. pisum*, *Aphis craccivora*, *Myzus persicae* and *Rhopalosiphum padi* [13,15]. The first Australian publication mentioning it was in 1978 following its discovery in pea germplasm imported from Sweden [16]. By 1988, PSbMV had been found in the states of Queensland (QLD), South Australia (SA) and Tasmania (TAS) [14,17], by 1998 in Western Australia (WA) and Victoria (VIC) [3], and by 2006 in New South Wales (NSW) [18]. Cool-season pulse species and four pasture legume species constitute the economically significant cultivated plants it infects in Australia [3,4,5]. In addition to infecting the pulses field pea, faba bean, chickpea and lentil in Australia [3,4,5,6,7,8,9,10,14,17,18,19,20], PSbMV also infects vegetable pea [21] and the minor pulses common vetch (*V. sativa*), narbon bean (*V. narbonensis*), purple vetch (*V. benghalensis*), bitter vetch (*V. ervilia*), grass pea (*Lathyrus sativus*), dwarf chickling (*L. cicera*), *L. clymenum*, *L. ochrus* and fenugreek (*Trigonella foenum-graecum*) [4,14,22]. However, it failed to infect all nine lupin species inoculated by Coutts et al. [5] and those listed in Table 1 of Volume 1 [1].

**Figure 1:**
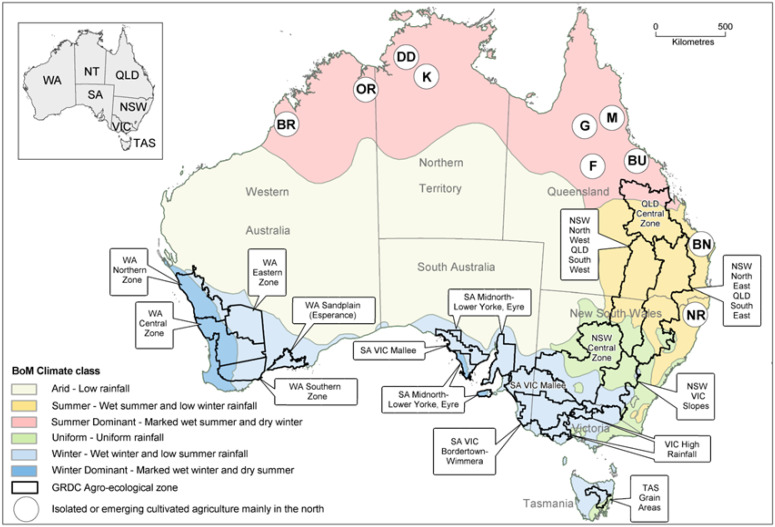
Map showing the main regions and additional smaller northern districts where pulses are grown within Australia. The State and Territory acronyms used are NT (Northern Territory), TAS (Tasmania), SA (South Australia), WA (Western Australia), VIC (Victoria), NSW (New South Wales) and QLD (Queensland). The smaller irrigated pulse-growing districts are NSW, NR, Northern Rivers; NT, DD, Douglas/Daly and K, Katherine; QLD, BU (Burdekin), BN (Bundaberg), G (Gilbert), F (Flinders) and M (Mareeba, including Atherton and Ravenshoe); and WA, BR (Broome) and OR (Ord River Irrigation Area). Different colors distinguish Australian Bureau of Meteorology (BOM) climate groupings. Boundaries of main agro-ecological zones are indicated with by black lines. Image credit@Department of Primary Industries and Regional Development/P. Goulding.

Figure 1 shows the Australian regions where PSbMV-susceptible pulse crops are planted. The main ones produced in the southern grainbelt (TAS, SA, and south and central south NSW) are field pea, lentil, faba bean and chickpea. Those in the northern grainbelt (central and southern QLD and north and central–north NSW) are field pea, lentil and chickpea [1]. In addition, there are other differences between regions, e.g., chickpea is not planted in TAS, central QLD does not plant faba bean or field pea, chickpea is planted under irrigation in tropical northern Australia, and all grain growing regions plant the PSbMV-susceptible and increasingly more widely grown minor pulse common vetch [23].

## 3. Disease Symptoms and Diagnostics

**Figure 2:**
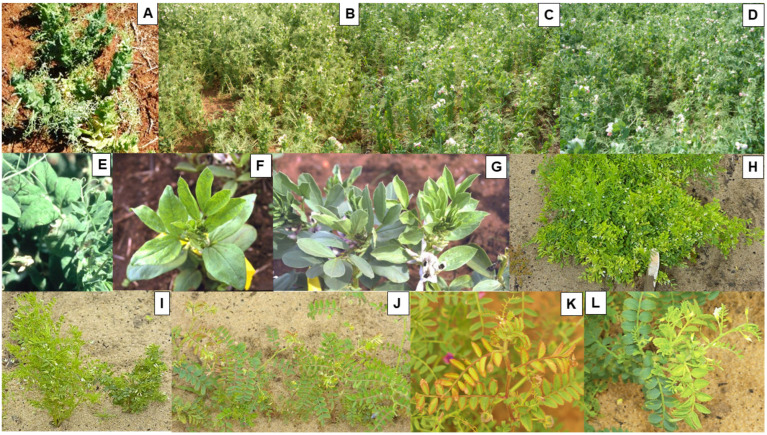
Disease symptoms of pea seed-borne mosaic (PSbMV) infection caused in foliage of the principal Australian cool-season pulse species. (**A**) Infected row of field pea (*Pisum sativum*) germplasm line with severe symptoms consisting of small bunched and deformed leaves and shoot dwarfing caused by infection with a virulent PSbMV strain (at Medina in 1997). (**B**) Plot of field pea cv. Kaspa showing infected plants with pale, reduced sized leaves and stunted growth (front) compared with healthy normal plants (back) (at Avondale in 2005). (**C**) Plot of field pea cv. Kaspa showing infected plants with stunting symptoms (at Avondale in 2005). (**D**) Plot of field pea cv. Kaspa showing small patch of infected plants with pale, reduced sized leaves and growth stunting symptoms compared with healthy normal plants surrounding them (at Avondale in 2005). (**E**). Infected shoot of field pea germplasm line showing leaf symptoms of severe mosaic and deformation caused by infection with a virulent PSbMV strain (at Medina in 1997). (**F**) Infected shoot of faba bean (*Vicia faba*) cv. Fiesta showing leaf symptoms of vein banding, mosaic, deformation and chlorosis (at Avondale in 1999). (**G**) Healthy plant of faba bean cv. Fiesta (left) compared with infected plant with leaf symptoms of mosaic, deformation and chlorosis (right) (at Avondale in 1999). (**H**) Partially infected row of lentil (*Lens culinaris*) plant breeding line showing leaf symptoms of mosaic, chlorosis and deformation (front) compared with healthy foliage growth (behind) (at Medina in 2004). (**I**) Plant of a lentil breeding line showing leaf symptoms of mosaic and deformation as well as plant stunting (right) compared with vigorously growing healthy plant (left) (at Medina in 2004). (**J**) Infected row of chickpea (*Cicer arietinum*) (Kabuli type) breeding line showing symptoms of leaflet pallor and deformation and twisting of apical shoot growth (at Medina in 2004). (**K**) Infected shoot of a chickpea breeding line (Desi type) infected with PSbMV, showing symptoms of tip distortion, mild mottle, chlorosis, and reddening (at Medina in 2003). (**L**) Infected shoot of chickpea cv. Almaz (Kabuli type) showing symptoms of leaf distortion, mild mottle, and chlorosis (at Medina in 2003). (**E**) Image credit@Department of Primary Industries and Regional Development/S. McKirdy; (**H**,**I**) modified from Coutts et al. [5].

**Figure 3:**
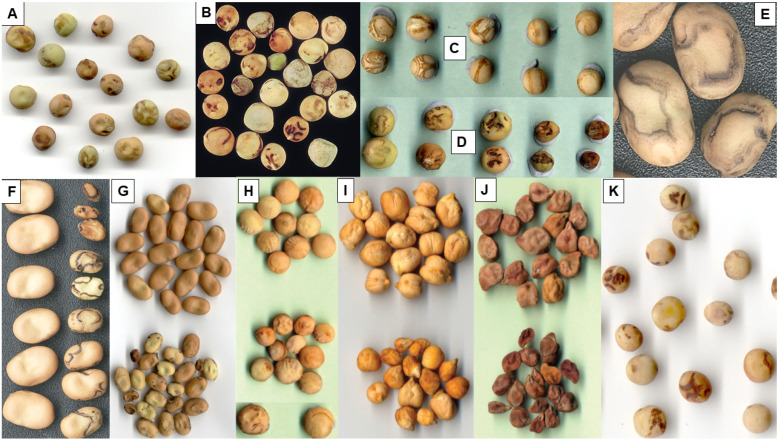
Disease symptoms of pea seed-borne mosaic (PSbMV) infection caused in seeds of the principal Australian cool-season pulse species. (**A**,**B**) Infected field pea (*Pisum sativum*) cv. Dundale seeds showing faint (**A**) or obvious (**B**) necrotic ring and line pattern seed coat symptoms (from Medina in 1999). (**C**) Infected seeds of field pea breeding line showing classic ‘tennis ball’-like or ‘blistering’ symptoms consisting of often concentric necrotic rings and circular cracks in their seed coats (from Medina in 2004). (**D**) Infected seeds of field pea breeding line showing seed coat symptoms of necrotic rings and lines, splitting, malformation and reduced size (from Medina in 2004). (**E**) Infected seeds of faba bean (*Vicia faba*) cv. Fiesta showing details of concentric necrotic rings in their seed coats (from Medina in 1999). (**F**) Infected seeds of faba bean (*Vicia faba*) cv. Fiesta showing necrotic rings and line patterns in their seed coats, malformation and size reduction (right) compared with larger, normal-looking healthy seeds (left) (from Medina in 1999). (**G**) Infected seeds of faba bean cv. unknown showing seed coat symptoms consisting of necrotic blotches and rings, shape malformation and size reduction (below) compared with larger, normal-looking healthy seeds (above) (from Medina in 1999). (**H**) Infected seeds of lentil (*Lens culinaris*) cv. Nipper showing seed coat symptoms of mild necrotic rings and line patterns and reduced size (below) compared with larger, healthy normal-looking seeds (above); two seeds at bottom show close-up view of necrotic rings (from Medina in 2003). (**I**) Infected seeds of chickpea (*Cicer arietinum*, Kabuli type) showing seed coat symptoms of faint necrotic rings and line patterns and reduced size (below) compared with larger, healthy normal-looking seeds (above) (from Medina in 2003). (**J**) Infected seeds of chickpea (Desi type) showing seed coat symptoms of faint necrotic rings and line patterns, reduced size, malformation and darkening (below) compared with larger, healthy normal-looking seeds (above) (from Medina in 2003). (**K**) Infected seeds of *Lathyrus ochrus* germplasm line showing seed coat symptoms of necrotic rings and line patterns, reduced size and malformation (from Medina in 1999). (**C**,**D**) Image credit7@Department of Primary Industries and Regional Development/R. Prince); (**B**,**F**) images modified from Latham and Jones [4]; (**H**–**J**) images modified from Coutts et al. [5]; (**E**,**G**) images modified from Makkouk et al. [26].

The symptoms PSbMV causes in infected cool-season pulse plants in Australia vary in type and severity. Its foliage symptoms include a wide range of different leaf, shoot and entire plant symptoms [3,4,5,7,24,25] (see also Table 1 in Volume 1 [1]). Table 1 of Volume 3 lists the principal foliage symptoms PSbMV causes in the main and minor cool-season pulses grown in Australia. However, when PSbMV was detected in symptomatic leaves from a fenugreek crop growing in SA, these leaves were also infected with other viruses, so which foliage symptoms it causes when present alone in this minor pulse is unknown [22]. Figure 2A–L provides Australian examples of the diverse range of symptom types that occur in the main pulses. In addition, PSbMV can also cause petal color break and both flower and pod distortion [13,25]. PSbMV-induced seed quality defects reported in cool-season pulses in Australia include necrotic rings, darkening malformation and cracking of seed coats, and such seeds are often small and shriveled (Figure 3A–K) [3,4,5,24,25,26]. The coats of seeds from PSbMV-infected chickpea plants were darkened [5]. Necrotic symptoms commonly occurred on the seed coats of faba bean (Figure 3E–G), grass pea, dwarf chickling and *L. ochrus* (Figure 3K), but less often on those of field pea, lentil and chickpea (Figure 3A–D,H–J). On the dark colored seed coats of common vetch, purple vetch and bitter vetch, they were less conspicuous [4]. Overall, amongst the major pulses, seed coat PSbMV symptom defects were most readily visible in the white seeded cultivars of field pea, faba bean and Kabuli chickpea [4,5]. In addition, the cotyledons of Kabuli chickpea seeds showed necrotic markings due to PSbMV infection, further compromising their value for human consumption [5]. Seed-to-seedling transmission was infrequent when seed samples of different cool-season pulse species with necrotic seed coat symptoms were sown, reflecting its association with mother plant pod infection rather than seed embryo infection [3,4,14,27].

Confirmation of PSbMV presence in field samples from symptomatic or asymptomatic cool-season pulse plants requires devising and utilizing effective diagnostic techniques. In SA studies published in 1991, Ligat et al. [14] employed sap and aphid inoculation to indicator hosts and enzyme-linked immunosorbent assay (ELISA) to detect PSbMV infection in leaf samples. They used gel immunodiffusion tests to establish whether serologically distinct PSbMV variants were present in leaves infected with different PSbMV isolates. They also modified the dot-immunobinding assay of Hibi and Saito [28] to provide a sensitive and fast-acting indirect dot-immunobinding assay employing nitrocellulose membranes for use in routine PSbMV detection in leaf samples. In SA studies published in 1993, Ligat and Randles [27] employed this dot-immunobinding assay to study PSbMV seed-to-seedling transmission in field pea. In SA studies published in 1998, Ali et al. [29] devised dot blot and tissue print hybridization assays for rapid detection of PSbMV in infected leaf and seed samples. They used radioactive (^32^P-labelled) and non-radioactive random primed cDNA probes covering PSbMV’s entire genome, both of which were equally effective regarding detection sensitivity. Moreover, PSbMV was detectable in leaf tissue squashes using cDNA probes in tissue-blot (=tissue-print) hybridizations. Tissue-blot hybridization with non-radioactive cDNA probes proved suitable for routine PSbMV detection in samples as non-specific hybridization was absent. In addition, this assay was suitable for use in the field without a requirement to transport samples to a suitably equipped laboratory prior to squashing onto membranes. However, rather than use cDNA probes, researchers in eastern Australia [8,10,18,19,30,31] adopted the tissue-blot immunoassay (TBIA) approach developed by the International Center for Agricultural Research in the Dry Areas (ICARDA) in Syria for routine testing of pulse leaf samples for virus infection, including for PSbMV [32]. It involves cutting petioles or leaf laminas with a razor blade, blotting their cut surfaces onto a nitrocellulose membrane, blocking against non-specific binding, then incubating with virus antibody followed by bound virus detection using a labelled secondary virus antibody. An advantage of TBIA over ELISA was that samples can be blotted individually for testing. This is instead of having to be grouped prior to testing. This is often needed with ELISA, which sometimes requires retesting at lower grouping levels before accurate percentage infection incidences can be established. However, TBIA also has a disadvantage in that interpreting tissue print results accurately can be problematic, especially when testing for phloem-limited viruses. For this reason, ELISA remained the preferred routine sample virus testing procedure in WA, including for PSbMV (e.g., Refs. [3,4,5,6,33]).

In an SA study published in 2001, Torok and Randles [34] developed a duplex reverse transcription–polymerase chain reaction (RT–PCR) assay with a tobacco mosaic virus (TMV; genus *Tobamovirus*, family *Virgaviridae*) internal control for the routine detection of PSbMV in pea seed samples. They concluded that this assay was suitable for use by quarantine authorities and plant breeders in large-scale screening of field pea germplasm for PSbMV. Research in SA published in 2007 devised an RT–PCR assay that depended upon an amplicon from the variable potyvirus HC-Pro coding region of viral RNA to differentiate between PSbMV and other potyviruses from legumes [35]. Later, Freeman et al. [8] compared the effectiveness of TBIA with that of RT–PCR for regular virus testing of samples from VIC and NSW pulse crops, including testing for PSbMV. When testing many samples, they concluded that TBIA was still the most appropriate procedure for obtaining accurate estimates of virus incidence, whereas RT–PCR was most useful for testing bulk samples when viruses are present at low incidence. A recent VIC study used targeted genome sequencing (TG-Seq) to detect PSbMV, BYMV, CMV and a fourth seed-borne pulse virus not yet found in Australia (pea early browning virus; genus *Tobravirus*, family *Virgaviridae*) within the same sample [36]. TG-Seq detected their amplicons within multiplex PCR reactions. This approach decreased the complexity of bioinformatics analysis and sequencing costs, and it was recommended for use with virus surveys in pulse crops and routine virus certification or biosecurity tests. A subsequent review focused on improving biosecurity against virus threats to Australian grain crops by incorporating high throughput sequencing (HTS) smart tools into sample testing procedures, and it used grain legume virus threats as examples [37]. The objective was to improve virus testing cost effectiveness, accuracy and efficiency using Illumina and Oxford Nanopore Technology shotgun sequencing, targeted viral genome HTS and virus vector metabarcoding. Their potential use extends beyond biosecurity and other plant health activities to enhance background virus and vector surveillance and management research activities, including those involving PSbMV and its variants when they infect cool-season pulses.

## 4. Transmission by Aphids, Contact and Seeds

**Figure 4:**
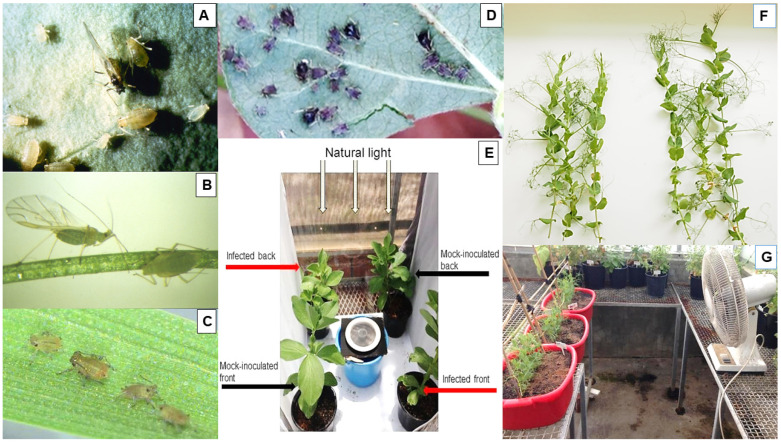
Transmission of pea seed-borne mosaic (PSbMV) by aphid and wind-mediated contact transmission in cool-season pulse species hosts. (**A**) Leaf lamina colonized by alate and apterous forms of major PSbMV vector aphid *Myzus persicae* (green peach aphid). (**B**) Leaf petiole colonized by alate and apterous forms of major PSbMV vector aphid *Acyrthosiphon kondoi* (blue green aphid). (**C**) Leaf lamina colonized by apterous forms of PSbMV vector aphid *Rhopalosiphum padi* (oat aphid). (**D**) Leaf lamina colonized by alate and apterous forms of PSbMV vector aphid *Aphis craccivora* (cowpea aphid). (**E**) Representation of a free-choice vector transmission assay within an aphid-rearing cage into which PSbMV-infected and mock-inoculated faba bean (*Vicia faba*) plants (two of each) were placed in each corner along with a centrally placed black cylindrical tube inserted into a black carboard platform containing 35 aphid alatae of the same aphid species ready for release. (**F**) Wind-mediated contact transmission study examining PSbMV spread in field pea (*Pisum sativum*) from a centrally placed infector plant to adjacent healthy plants growing with or without being blown by a fan: both adjacent plants stunted due to infection with blowing (left) or remained healthy without blowing (right). (**G**) Wind-mediated contact transmission studying PSbMV spread in field pea from a centrally placed infector plant to adjacent healthy plants in the presence or absence of blowing using oscillating pedestal fans to simulate wind. (**B**,**C**) Image credit@Department of Primary Industries and Rural Affairs/D. Thackray; (**D**) image modified from Clarke et al. [38]; (**E**) image modified from Congdon et al. [15]; (**F**,**G**) images modified from Congdon et al. [39].

A study from SA published in 1991 by Ligat et al. [14] reported that the two aphid species they used in aphid inoculation, *M. persicae* and *A. pisum*, both transmitted PSbMV readily. A more recent study by Congdon et al. [15] used four aphid species commonly found landing in WA field pea stands in which PSbMV was spreading: *M. persicae*, *Lipaphis pseudobrassicae*, *R. padi* and *A. kondoi* [6]. When their transmission efficiencies, and those of *Aphis craccivora*, were measured using PSbMV isolate W1 (in pathotype 2) and field pea cv. Kaspa, they were 27% (*Aphis craccivora*), 26% (*M. persicae*), 6% (*A. kondoi*) and 3% (*R. padi*), respectively, (Figure 4A–D) but *L. pseudobrassicae* was a non-vector [15]. When these transmission efficiency studies were repeated with *M. persicae*, *Aphis craccivora* and *R. padi,* and plants of field pea cv. Twilight, which has partial resistance to aphid inoculation, the PSbMV transmission efficiencies obtained were diminished to 16% (*M. persicae*), 12% (*Aphis craccivora*) and 1% (*R. padi*). Amongst these aphid vector species, *R. padi* was still likely to be important because its population often reaches very high numbers in the immediate vicinity of field pea crops despite it being an inefficient vector that does not colonize pulses. When free-choice assays were done to study the alighting preferences of winged *M. persicae* and *R. padi* upon field pea plants (Figure 4E), *M. persicae* alighted on mock inoculated (=healthy) plants preferentially for up to 24 h whereas *R. padi* preferred to land on PSbMV-infected plants for up to 4 h, eventually switching their landing preference to healthy plants after 48 h. There was an increase in numbers of volatile compounds (such as esters, ketones and aldehydes) above PSbMV-infected plants compared to above healthy plants. When this study was repeated with faba bean plants in place of field pea plants, the results were similar but less clear cut. These findings suggested manipulation by PSbMV to alter the emission profile of volatile compounds, which possibly caused the differences in aphid alighting preferences. A preference to land first on infected plants and then on healthy plants, as with *R. padi,* would enhance spread, especially in the early phase of a PSbMV epidemic.

Another WA study with PSbMV examined its stability in infective sap and its wind-mediated contact transmission from infected to healthy field pea plants of cvs Kaspa and Twilight [39]. When infective leaf sap was kept at room temperature, PSbMV isolate W1 remained highly infective after 6 h and was still infective at low levels after 30 h. When leaves of infected and healthy plants were rubbed against each other, the leaf surface wounding caused by this direct contact enabled PSbMV transmission from infected to healthy plants. Such transmission also occurred when intertwined healthy and PSbMV-infected plants were blown against by simulated wind generated by fans (Figure 4F,G). This wind-mediated contact transmission occurred consistently when the plants were kept at 14–20 °C and was enhanced by dusting the plants beforehand with diatomaceous earth, simulating damage by sand or dust particle blasting of foliage caused by wind currents. However, keeping the plants at 20–30 °C instead of 14–20 °C greatly diminished its transmission to heathy plants. Therefore, when wind-mediated wounding occurs in field situations where PSbMV-infected plants touch healthy plants, its contact transmission is likely to take place. Should this transmission occur before aphid vector arrival in pulse crops, the resulting early expansion of initial crop infection foci around seed-infected plants during the critical initial phase of a PSbMV epidemic has the potential to increase the spread of the virus significantly. This would be likely to result in enhanced seed yield and quality losses and infection of harvested seed. Although there is anecdotal evidence that some PSbMV spread can occur when no aphids are present, field experiments are needed to establish whether wind-mediated contact transmission is a significant contributor to PSbMV spread in commercial crops of field pea and other pulses.

An SA study published in 1991 examined the relationship between seed size and rate of PSbMV seed transmission [14]. Five PSbMV isolates (US, Q, S4, S6, T) were inoculated to plants of field pea cv. Dundale and the seeds produced by the infected plants were harvested. The seed was sub-divided into fractions of different sizes by differential sieving before being weighed and then sown prior to testing the seedlings individually for PSbMV. Seed transmission rates varied depending upon seed size, being lowest in the smallest (27–40%) and highest in the biggest (82–91%) seed fractions. Regardless of seed fraction size, seed from healthy plants always weighed more and had a higher germination rate than seed from PSbMV-infected plants. Also, germination was always poorest in the smallest seed fraction. Also in SA, seed transmission in cv. Dundale was studied over five seedling generations using four of the same PSbMV isolates (US, Q, S4 and S6) [27]. With all four isolates, the virus reached high concentrations, and symptoms were present in foliage up to the second generation in the infected seedlings produced. By the third, fourth and fifth generations, however, virus concentration was always low and infection was asymptomatic in seedling foliage. By contrast, in all five seedling generations, the virus reached high concentrations in the cotyledon, testa and embryo of mature seed and in floral tissues. Nonetheless, PSbMV seed transmission to seedlings only occurred with embryo infection. Thus, these findings revealed that, despite being subliminal in foliage after the second seedling generation, PSbMV infection still reaches high levels in floral parts and seeds, resulting in high rates of transmission to seedlings [27]. Further Australian studies providing information on PSbMV seed-to-seedling transmission in field pea and other cool-season pulses are covered in Section 5, Section 6, Section 7, Section 8 and Section 9 below.

## 5. Occurrence in Crops and Seed Stocks

In 1984 and 1985, PSbMV was detected in 12/83 seed samples from commercial field pea crops from the southeast, mid-north, Adelaide and York Peninsula regions of SA [14]. In 1994–1999, surveying experimental plots containing major pulses in south-west WA found PSbMV infecting symptomatic plants of field pea, faba bean and lentil, but not in chickpea or the minor pulses narbon bean, grass pea, dwarf chickling, *L. clymenum* or fenugreek [3,4]. In 1998 and 1999, larger-scale surveys of commercial field pea and faba bean crops growing in the same region found the proportion of crops infected with PSbMV within any one year was up to 42% for field pea, but only 3% in faba bean. However, the within-crop incidence was generally low, not exceeding 9% in field pea or 2% in faba bean. Also, tests on commercial WA seed stocks from 1998 and 1999 found PSbMV in 11/30 (0.1–10% seed transmission) field pea, 1/11 (0.2% seed transmission) faba bean and 1/50 (0.1% seed transmission) chickpea samples, and a field pea seed sample from VIC tested in WA was 0.1% PSbMV-infected [3]. In 2003 and 2004, when 116 different seed lots from experimental plots growing at seven WA sites were examined for quality defects due to PSbMV infection, these were present in all those of Kabuli chickpea (5) and field pea (70), and in 10/18 of faba bean, but in none of lentil (23). Seedlings from 23 of these field pea and faba bean seed lots were tested for presence of PSbMV and two other viruses known to cause pulse seed defects elsewhere: broad bean true mosaic virus (BBTMV) and broad bean stain virus (BBSV), both in genus *Comovirus*, family *Secoviridae* [26,40]. Seed-borne PSbMV was found in 5/7 (<1–14% incidence) field pea and 0/16 (0% incidence) faba bean seed lots. Neither BBTV nor BBSV were detected [5]. In 2007, when seedlings from 45 commercial field pea seed stock samples of cvs Parafield (12) and Kaspa (33) were tested, seed-borne PSbMV was detected in all of them [5]. The PSbMV infection incidences found in cvs Parafield and Kaspa were 0.3–30% and 0.5–47%, respectively. From 2013 to 2015, surveys for PSbMV in commercial field pea crops and experimental plots in south-west WA found it in 9/31 crops at 2–51% incidences, 20/24 cultivar plots at 1–100% incidences and 14/21 breeding line plots at 1–57% incidences [9]. Both crops and plots of cvs Gunyah, Kaspa and Twilight were infected frequently, but no infection was found in cv. Wharton in which PSbMV resistance gene *sbm1* is present. Thus, with the exception of a cultivar with *sbm1*, PSbMV infection was widespread in field pea in WA over the 17-year period (1998–2015) when these surveys of commercial crops, experimental plots and seed stocks were done.

In 2006, a survey of field pea crops from southern NSW and VIC, and faba bean crops from NSW, detected PSbMV in (i) 12/21 field pea crops from NSW at incidences of 32–77%; (ii) 2/10 field pea crops from VIC at incidences of 1–2%; and (iii) 1/3 faba bean crops from NSW at an incidence of 1% [8]. In six NSW field pea seed lots, PSbMV seedling infection was 0–23%. In 2012, seedlings from 5/24 lentil accessions imported from the USDA lentil collection were found to be PSbMV-infected, demonstrating the presence of seed-borne PSbMV in this host [19]. The PSbMV seed infection incidences were between 1 and 10%. In 2000–2007, annual surveys of lentil, faba bean and chickpea crops in VIC and SA sometimes detected PSbMV but were only published in brief conference abstract format, so details of the extent of crop infection are no longer accessible [41,42,43,44,45]. In a study published in 2025, field pea seed samples collected in NSW, WA and VIC in 2005–2010 were tested for PSbMV seed-to-seedling transmission [10]. PSbMV was detected in 58/92 (63%) of samples from commercial fields and 34/52 (65%) from field plots. The rates of seed-to-seedling transmission found were (numbers infected in parentheses), for commercial field samples, 0% (34), 1–5% (29), >5–10% (19) and >10% (10), and, for field plots, 0% (18), 1–5% (16), >5–10% (8) and >10% (10). Cvs Kaspa and Excel were most commonly affected of the 29 cultivars tested, and overall levels of seed transmission were greater in seed samples from field plots than from commercial fields [10].

## 6. Pathotypes and Genetic Diversity

In overseas studies, the range of different phenotypes that resulted when PSbMV isolates were inoculated to differential genotypes of field pea allowed the separation of PSbMV isolates into different strain groups or pathotypes. Initially, four PSbMV strain groups called I, II, III and IV were identified based on the type of host response that developed [46]. Soon afterwards, three pathotypes were identified called P-1, P-2 (sometimes called lentil strain L1), and P-4 [46,47,48,49,50,51]; confirmation of the existence of previously suspected pathotype P-3 was reported later [52]. The different phenotypes that resulted when pathotypes P-1, P-2 and P-4 were inoculated to field pea differential lines suggested the existence of four resistance genes: *sbm-1*, *sbm-2*, *sbm-3* and *sbm-4* [53]. Initially, it was thought that *sbm-1* conferred resistance to P-1, *sbm-2* and *sbm-3* conferred resistance to P-2, and *sbm-4* conferred resistance to P-4 [53]. However, Johansen et al. [54] later found that the four pathotypes of PSbMV could be deduced from their interactions with just two cistrons, and, soon afterwards, Gao et al. [55] found evidence for the existence of only two resistance genes *sbm-1* and *sbm-2*. Thus, *sbm-3* and *sbm-4* were non-existent. Resistance to all four pathotypes was conferred by *sbm-1*, its allele *sbm-1^1^* conferred resistance to P-1 and P-2, and *sbm-2* conferred resistance to P-2 and P-3. Thus, P-1 differed from P-2 and P-3 in overcoming *sbm-2*, P-3 differed from P-2 in overcoming allele *sbm-1^1^*, and P-4 differed from P-1, P-2 and P-3 in overcoming both allele *sbm-1^1^* and *sbm-2* [26,55]. In SA research by Ali and Randles, two more pathotypes, U-1 and U-2, were revealed when Pakistani PSbMV isolates were inoculated to standard field pea differentials [24].

In SA studies published in 1993, in which Australian PSbMV isolates were inoculated to field pea differentials, Ligat and Randles [27] identified isolate Q as belonging to pathotype P-1 and isolates S4 and S6 as belonging to P-4. Research in SA published in 1998 studied the effects of sap-inoculating two PSbMV isolates (S6 and US) belonging to pathotypes P-1 (US) and P-4 (S6) to plants of field pea cv. Dundale, including symptom development, seed yield and seed infection rate [25]. The P-4 isolate elicited a sensitive phenotype that included foliage symptoms of severe mosaic, leaf deformation, plant stunting, and delayed both flowering and pod development, whereas the P-1 isolate elicited a more tolerant phenotype in which foliage symptoms of leaf rolling and plant stunting were transient. Seed yield was diminished by 82% with P-4 infection but only by 31% with P-1 infection. PSbMV infection of seedlings grown from harvested seed reached 31% with the P-4 isolate but only 8% with the P-1 isolate. Also, when seed-infected plants were grown, infection with the P-4 isolate reduced seed yield by 52% and infected 12% of seedlings grown from harvested seed. By contrast, although P-1 infected 7% of seedlings, it failed to decrease seed yield significantly. Thus, the more virulent P-4 isolate differed from the milder P-1 isolate by causing more severe symptoms, greater seed yield losses and higher infection levels in seedlings grown from harvested seed [25].

In 1997, PSbMV-infected plants with severe foliage symptoms were found growing within field pea breeding plots in WA (Figure 2A,E) [3]. The PSbMV isolate (W1) obtained from these plants belonged to pathotype P-2 [5,56]. In 2005, PSbMV isolate W1 infector plants were introduced into field plots of lentil cv. Nugget in WA. Naturally occurring winged aphid vectors spread PSbMV to healthy lentil plants [5]. Symptomatic current-season infected lentil plants within plots with infector plants and asymptomatic plants in plots without infector plants were tagged near to the end of the growing season. Leaf samples from each tagged plant were tested for PSbMV by ELISA to confirm the visual diagnoses. PSbMV infection diminished shoot dry weight, seed yield, seed number/plant and individual seed weight by 23%, 96%, 90% and 58%, respectively. A PSbMV seed transmission rate to seedlings of 6% was detected in seeds from the infected lentil plants [5]. In SA studies published in 2007, in which Australian PSbMV isolates were inoculated to field pea differentials, Torok and Randles [35] classified isolates obtained in 1985–1987 from field pea in SA, VIC or WA as follows: three isolates as pathotype P-1, and 11 isolates, which included S4 and S6, as pathotype P-4. In NSW studies published in 2013, 24 seed samples of lentil accessions imported into Australia from the USDA germplasm collection, where they had been reported to be PSbMV-contaminated [57,58], were obtained from the Australian Temperate Field Crop Germplasm Collection [19]. When they were tested for seed-borne PSbMV infection, it was detected in five of them at seed-to-seedling transmission rates of 1–10%. These infected lentil seedlings provided 20 PSbMV isolates, 16 of which were pathotyped by inoculation to field pea differentials. All 16 lentil isolates belonged to pathotype P-2, which had previously been called lentil pathotype L-1 by Alconero et al. [47]. At that time, the only Australian report of PSbMV seed-to-seedling transmission for field-grown lentil was the 6% seed transmission record mentioned earlier in this paragraph for lentil plants in WA infected with pathotype P-2 isolate W1, originally from field pea [5]. Subsequently, in research published in 2025, when 71 PSbMV isolates obtained in 2005–2010 from 15 PSbMV-infected field pea seed lots from NSW, VIC or WA were pathotyped using field pea differentials, all four PSbMV pathotypes were found [10]. These belonged to pathotypes P-1 (8 isolates), P-2 (1 isolate), P-3 (42 isolates) and P-4 (20 isolates). Thus, P-2 was the least common pathotype and P-3 was the most common, its presence not having been revealed in Australia previously because earlier Australian pathotyping studies had omitted including a reliable *sbm2* differential host that would disclose its presence. In addition, the second PSbMV isolate belonging to pathotype P-2 from field pea was found, this time from cv. Dundale from NSW. Whether the latter isolate was as virulent as pathotype P-2 isolate W1 from WA [5] and pathotype P-2 isolate L1 from lentil in the USA [47,48,51,57] was not investigated. Pathotype P-2 isolate Pam from field pea cv. Pamaro in New Zealand was less virulent than both P-2 isolate L1 from the USA and virulent pathotype P-2 isolate E210 from field pea in the Netherlands [59]. Thus, P-2 isolates differ in virulence, and virulent field pea isolates W1 and E210 resemble lentil isolate L1 from lentil in their virulence, including in lentil in which W1 caused a 96% yield loss (mentioned earlier in this paragraph).

In research in SA published in 2007, when the helper component proteinase (HCPro) RT–PCR products from 28 PSbMV isolates, which included 14 obtained between 1985 and 1997 in Australia (12 from SA, 1 each from VIC and WA), 13 from Pakistan and one from the USA, were separated following restriction endonuclease treatment (REN), eight different REN treatment groupings were found, whereas phylogenetic analysis placed these same isolates into three phylogroups [35]. The Australian isolates fitted within 3/8 REN groups and 2/3 unnamed phylogroups. They belonged to pathotypes P-1 or P-4, the USA isolate (L1) to pathotype P-2, and the Pakistani isolates to pathotypes P-1, P-4, U-1, and U-2, and an undetermined pathotype. However, there was no clear linkage between PSbMV isolate geographical origin or pathotype and these REN groupings or any phylogroups [35]. A subsequent study in WA published in 2011 compared the complete or partial coat protein (CP) sequences of 11 Australian PSbMV isolates from NSW, VIC or WA with those of 21 others from different countries [56]. On phylogenetic analysis, these 32 isolates fitted within three phylogroups (A–C), with Australian isolates being present in A and C but not in monotypic phylogroup B, which only contained USA lentil isolate L1. In addition, within phylogroup A, there were three subclades (Ai, Aii and Aiii), two of which contained Australian isolates (Ai and Aiii). Isolates belonging to pathotype P-1 fitted with phylogroup A (either in Ai or Aii), whereas the P-4 isolates were all within phylogroup C. By contrast, the two P-2 isolates fitted within different phylogroups: W1 from Australian field pea within phylogroup A (in Ai) and L1 from lentil in the USA on its own within monophyletic phylogroup B. Isolate PK9 from phylogroup U-2 was within phylogroup A (in Ai). A study published in 2015 found that the CP sequence of PSbMV isolate GR33 from lentil growing in Greece fitted within minor phylogroup Aii rather than with lentil isolate L1 within phylogroup B [60].

In 2015, 14 new PSbMV isolates from various locations in the high medium and low rainfall zones of the WA grainbelt were tested by RT–PCR [9]. By sequencing their amplicons, partial CP sequences were obtained. These 14 new partial CP sequences were compared with 12 other CPs from Australia and 27 others from four other continents obtained from GenBank. Again, the Australian isolates all fitted within phylogroup A minor phylogroups Ai and Aiii, or within phylogroup C. Thus, from both studies there was evidence for at least three separate PSbMV incursions into the Australian continent. In addition to the pathotype P-2 isolates W1 from field pea and L1 from lentil [56], the isolates recognized by Congdon et al. [9] as belonging to P-2 included Chinese isolate China1 from faba bean and Australian isolate 7-6.19 from field pea in NSW, both of which Wylie et al. [56] had included in analyses without specifying their pathotype. These four P-2 isolates fitted within either phylogroup A (W1 in Ai, China1 in Aii and 7-6.19 in Aiii) or phylogroup B (L1). In a 2021 report, PSbMV was isolated from a single field pea sample from VIC and a complete genomic sequence was obtained [36]. In a study reported in 2025, Ademe et al. [61] compared the CP sequences from PCR amplicons they obtained from nine Ethiopian PSbMV isolates from lentil (8) or chickpea (1) with 29 others from GenBank, including six from Australia. They interpreted their phylogenetic analysis as revealing the presence of four distinct major phylogroups, which they named A, B, C and D. They also subdivided A into minor phylogroups AI and AII and B into BI and BII. Except with major phylogroup B, their naming system differed from that of Wylie et al. [56] as their phylogroup A corresponded with phylogroup C, their phylogroup C with minor phylogroup Aii, and their phylogroup D with minor phylogroups Ai and Aiii. Australian isolates 7-4.19 and 74.20 from NSW fitted within their phylogroup D whereas Australian isolates 7-4.22, 7-4.26, 7-4.33 and 5-22.35 from WA fitted within their minor phylogroup AI. Five of their eight Ethiopian isolates from lentil, along with their isolate from chickpea, fitted within their minor phylogroup BI, and two of them fitted within their phylogroup D. Their Ethiopian lentil isolate EthLe343-18 was in their minor phylogroup BII, which contained just two other isolates: field pea isolate DSMZ PV-0302 from Germany and pathotype P-2 lentil isolate L1 from the USA. The two other pathotype P-2 isolates they included in their analysis were placed in their phylogroups C (China1 from faba bean) and, as mentioned above, in phylogroup D (7-6.19 from Australian field pea). Pathotype P-1 isolates were within phylogroups C or D, whereas P-4 isolates were all within phylogroup AI.

Unless safeguarded against rigorously, the inadvertent inclusion of infected pulse seed within national breeding trials dispersed around Australia and the unintended release of new cultivars with seed-borne infection are likely to spread new PSbMV variants around Australia. Taking all the findings for PSbMV isolate virulence and the PSbMV phylogenetic studies described above together, they show that pathotypes P-1, P-2, P-3 and P-4 all occur in Australia, but pathotypes U-1 and U-2 have not yet spread here despite first being studied in SA. However, they provide no support for the existence of a distinct severe pathotype P-2 strain exclusively infecting lentil (i.e., isolate L1) suggested previously by several authors [10,19,48,51,57]. This is because some P-2 isolates from field pea, e.g., WI and E210, also caused severe symptoms, including in lentil, and isolate W1 was readily seed-borne in lentil. Furthermore, using the Wylie et al. [56] phylogroup classification system, Australian isolates are present within minor phylogroups Ai and Aiii, and phylogroup C, but none as yet within minor phylogroup Aii or phylogroup B. Therefore, at least three incursions have occurred but future incursions to the continent could introduce isolates belonging to Ai and B. More generally, these PSbMV studies found no significant relationship between the narrow phenotypic category of pathotype and phylogenetic placement. For example, both pathotype P-1 and P-2 isolates and isolates from lentil fitted within more than one phylogroup.

Additional Australian PSbMV genomic sequences are needed from different cool-season pulse species and regions to provide a more comprehensive picture of the genetic variability of this virus within Australia as well as where PSbMV originated and spread from to eventually reach the Australian continent. Furthermore, more biological data are required to understand the phenotypic diversity of PSbMV isolates beyond their pathotype (see Section 7 below), including host and vector ranges and affinities, intrinsic vertical and horizontal transmissibility, and virulence in both susceptible and partially resistant hosts. This data taken together with viral genomic data would provide not only a better understanding of the PSbMV species itself, but also a foundation for assessing the relative importance of different strains and potential biosecurity threats to future pulse production.

## 7. Host Resistance and Alternative Hosts

In a glasshouse study in SA published in 1991, five PSbMV isolates (US, S4, S6, Q, T) were sap- and aphid-inoculated to 16 field pea cultivars and breeding lines, 21 breeding lines of lentil, dwarf chickling, grass pea or *L. ochrus*, and single cultivars each of common bean and faba bean [14]. With few exceptions, all became PSbMV-infected. The exceptions were that common bean was a non-host to all five isolates; one isolate (T) failed to infect dwarf chickling, grass pea and *L. ochrus*; another isolate (S) was the only one to infect field pea cv. Greenfeast, which apparently had strain-specific PSbMV resistance; and aphid inoculation failed to infect field pea cv. Maitland. Symptom severity varied between different isolates.

**Figure 5:**
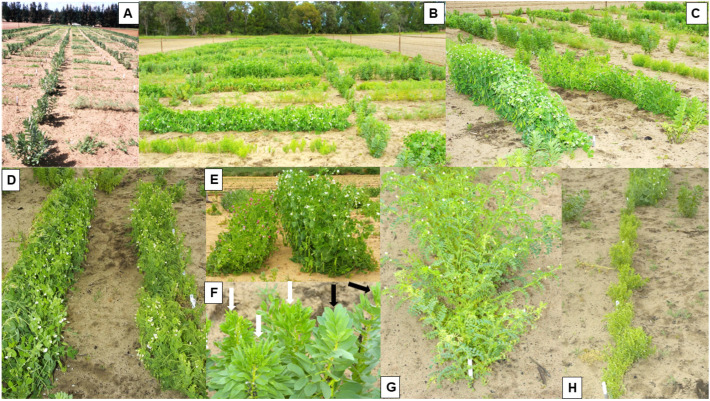
Screening for resistance to pea seed-borne mosaic (PSbMV) in cool-season pulses and pasture legumes. (**A**) Different pulse and pasture legume species being screened for PSbMV resistance using replicated (three times) and short (1.5 m length) single-row plots sown with cultivars, breeding lines and germplasm accessions. Faba bean (*Vicia faba*) transplants infected with PSbMV isolate WI were positioned at both ends of each test row, providing a uniform inoculum source for its spread by naturally occurring aphids (at Medina in 1998). (**B**) Different cool-season pulse species being screened for PSbMV resistance using replicated (six times) and longer (3 m length) single-row plots sown with cultivars, breeding lines and germplasm accessions of faba bean, field pea (*Pisum sativum*), lentil (*Lens culinaris*) and chickpea (*Cicer arietiunum*). Faba bean transplants infected with PSbMV isolate WI were positioned in between the ends of adjacent test rows (at Medina in 2004). (**C**) Later general view of some plots shown in (**B**): corner vigorously growing field pea test row PSbMV resistant (left) compared with adjacent infected field pea test row with chlorotic stunted plants (right); rows of lentil, chickpea or faba bean (behind). (**D**) Infected row of field pea showing chlorotic partially stunted plants (right) compared with normal-looking PSbMV resistant row without symptoms (left) (at Medina in 2004). (**E**) Infected row of field pea cv. Parafield plants with pale green, chlorotic foliage and severe stunting (left) compared with row with vigorously growing plants of PSbMV-resistant accession PI193586 without symptoms (right) (from Medina in 2004) (**F**) Partially infected row of faba bean containing healthy plants (black arrows) and infected plants with symptoms of leaf mosaic, deformation and size reduction (white arrows) (from Medina in 2004). (**G**) Infected row of Kabili chickpea showing apical growth symptoms of leaf pallor and bent shoots (at Medina in 2004). (**H**) Infected lentil row consisting of highly stunted plants with small pale leaves (at Medina in 2004). (**E**) Image modified from Coutts et al. [5].

In 1999, a field experiment in WA examined the susceptibilities and sensitivities of pulses other than lupins to PSbMV infection (Figure 5A) [4]. The susceptibility ranking system of McKirdy et al. [62] was used, accompanied by ELISA tests to confirm PSbMV presence in tip leaf samples from plants with distinct symptom types and where symptoms were unclear or absent. Sensitivity rankings were based on the intensity (i.e., severity) of the PSbMV symptoms that developed. The 17 field pea, five narbon bean, and three dwarf chickling genotypes tested all received highly susceptible, susceptible, or moderately resistant rankings. By contrast, the six faba bean, three common vetch, three *L. ochrus*, two purple vetch and single bitter vetch genotypes were all moderately resistant or resistant. The six genotypes each of chickpea and lentil were all resistant (=possess a form of partial resistance where very few plants became infected), whilst, amongst the three grass pea genotypes, two were ranked as resistant and one as highly resistant (=no plants became infected). Sensitivity rankings varied widely from high in some narbon bean and dwarf chickling genotypes to low in some field pea genotypes. PSbMV seed transmission to seedlings was found in field pea (1–18%), faba bean (2%), common vetch (0.3%), purple vetch (0.1%), grass pea (1%), dwarf chickling (0.4%), *L. clymenum* (5%) and *L. ochrus* (0.7%). In addition, the seed quality defect consisting predominantly of necrotic ring markings was visible on seeds of nine pulse species (field pea, faba bean, chickpea, dwarf chickling, grass pea, *L. ochrus*, common vetch, bitter vetch and purple vetch) (Figure 3A,B,E,G,K). They were most frequent in seeds of faba bean, dwarf chickling, grass pea and *L. ochrus*. There were insufficient seeds of lentil and narbon bean to assess for seed transmission or seed defects.

Potential alternative PSbMV hosts, consisting of 18 cultivars or genotypes belonging to five genera of pasture or forage legumes (1 per species) grown in Australia [63,64], were also evaluated within the 1999 study described in the previous paragraph [4]. These were (numbers of genotypes in parentheses): subterranean clover (*Trifolium subterraneum*) and other clover species (*Trifolium* spp.) (14), yellow serradella (*Ornithopus compressus*) (1), biserrula (*Biserrula pelecinus*) (1), sulla (*Hedysarum coronarium*) (1) and trigonella (*Trigonella balansae*) (1). A low level of asymptomatic infection (<5% of plants) classed as resistance was found in five species: eastern star clover (*T. dasyurum*), Persian clover (*T. resupinatum*), sea clover (*T. squarrosum*) and trigonella, but none of the genotypes or cultivars of the 14 other species tested became infected.

In 2003–2005, three further field experiments in WA examined the phenotypic responses of different genotypes of field pea, lentil, faba bean and chickpea, and single genotypes each of seven lupin species, to infection with PSbMV isolate W1 (Figure 5B–H) [5]. These field experiments differed from those of Latham and Jones [4] in that, instead of relying upon visual assessment for PSbMV symptoms, large-scale ELISA testing of leaf samples was used from each single-row plot to establish susceptibility and sensitivity rankings. Amongst the 39 field pea genotypes evaluated, 25 were highly susceptible or susceptible, nine were moderately resistant and five (WAPEA2128, WAPEA2140, 96-29, PI193586, and cv. Yarrum) were ranked as highly resistant (Figure 5C–E). Cv. Yarrum is known to carry PSbMV resistance gene *sbm1* [9]. All seven lupin species evaluated (narrow-leafed lupin cv. Tanjil, white lupin cv. Kiev Mutant, yellow lupin cv. Wodjil, pearl lupin P26956, *L. atlanticus* 93E002-4-11-7, *L. cosentinii* P20846 and *L. pilosus* 97P008-1) were highly resistant [5]. When the five field pea genotypes and seven lupin species ranked highly resistant, and two other lupin species (*L. hispanicus* P22982, *L. digitatus* P27045), were sap-inoculated and graft-inoculated with PSbMV isolates W1 and W2 in glasshouse tests, none of the lupin plants became infected. The susceptibility rankings for the other three pulse species were 14/14 highly susceptible for faba bean, 12/16 highly susceptible, and 4/16 susceptible for lentil, and 7/57 susceptible but 50/57 moderately resistant for chickpea. Foliage sensitivity rankings once infected varied between different genotypes, ranging from highly sensitive to sensitive in faba bean and Kabuli chickpea, sensitive to moderately sensitive in Desi chickpea, moderately sensitive to tolerant with field pea, and mostly tolerant with lentil (Figure 5F–H). Figure 2H–L show details of PSbMV foliage symptoms in plants of lentil and chickpea growing within these PSbMV-screening experiments (at Medina in 2003 and 2004). Seed quality defects were severe in seeds harvested from PSbMV-infected plants of field pea, chickpea and faba bean (Figure 3C,D), but milder in lentil seeds (Figure 3H). In addition to necrotic rings and line patterns on the seed coat, they consisted of splitting, malformation and diminished size (Figure 3C,D,H,I). Seed coat darkening was also evident with chickpea (Figure 3I,J).

The research findings described above in this Section emphasize the importance of not releasing new field pea or other cool-season pulse cultivars with high susceptibilities and sensitivities to PSbMV infection from grain legume breeding programs. This includes avoiding releasing new cultivars that readily develop seed defects caused by PSbMV infection or have high intrinsic PSbMV seed transmission rates. They also emphasize the need to avoid releasing PSbMV-contaminated seed of new cultivars. In addition, they also suggest that pasture and forage legumes are unlikely to act as significant alternative host PSbMV reservoirs for its spread to Australian cool-season pulse crops.

As one of its priorities, the Australian national field pea breeding program includes screening of its early-generation genotypes for PSbMV resistance and breeding new cultivars with this resistance [18,19]. In 2006–2009, in the Liverpool Plains region of northern NSW, a field experiment with single-row plots and two replicates screened Australian field pea cultivars, and both advanced generation and early generation breeding lines for PSbMV resistance [19]. PSbMV was introduced by sowing spreader rows with seed from a mixture of naturally PSbMV-infected seed lots in which pathotype P-4 was predominant but pathotype P-1 was also present. The numbers of different field pea genotypes evaluated were 200 (2006), 252 (2007), 296 (2008) and 222 (2009). Leaf samples from each row were collected at random (15–20 plants/row) and tested for PSbMV by TBIA, which provided PSbMV incidence data relevant to host resistance. Symptom scores were made twice per year, and seed yield data were obtained for each genotype. From genotypes of interest, harvested seed was tested for PSbMV transmission to seedlings, and plants were evaluated by sap inoculation with a pathotype P-4 isolate in the glasshouse. Cultivars Yarrum, Maki and Walana were the only ones with resistance gene *sbm1*. However, since these cultivars were impure, when released, each of them still produced a small proportion of plants lacking this resistance gene. Breeding lines G-1000 and OZP805 behaved like cvs Yarrum, Maki and Walana so carried gene *sbm1*; OZP805 was released as PSbMV-resistant cv. Wharton. Cultivars Glenroy and Soupa were consistent in having partial resistance to PSbMV as its incidence was low in them each year. Amongst the other cultivars evaluated, only cvs Cressy Blue, Boreen and Mukta had consistently low PSbMV seed transmission rates. Instead of breeding for partial PSbMV resistance, low PSbMV seed transmission rates or incorporating PSbMV resistance gene *sbm2*, this study recommended focusing on incorporating gene *sbm1* within all new cultivars released by the Australian national field pea breeding program. This was because it was effective against pathotypes P-1 to P-4 [10,19]. In the future, however, this recommendation might need reconsideration should pathotypes U-1 or U-2 arrive in Australia.

In a study in WA published in 2016, plants of two field pea cultivars without resistance, two with resistance gene *sbm1* (Wharton and Yarrum), and another two with resistance gene *sbm2* (Greenfeast and Gunyah), were sap-inoculated with PSbMV pathotype P-2 isolate W1 and unpathotyped isolate ‘Kaspa’ derived from infected field pea cv. Kaspa seed [9]. Whilst both isolates readily infected the two susceptible cultivars, isolate ‘Kaspa’ was unable to overcome any of the resistant cultivars, whilst isolate W1 infected a small number of plants of each of the *sbm1*- and *sbm2*-carrying cultivars. This suggested (i) the presence of a low level of resistance-breaking PSbMV variants within the isolate W1 culture but not within the ‘Kaspa’ culture; or (ii) the impurity of the seed stocks of the *sbm1*- and *sbm2*-carrying cultivars tested. This study also identified partial resistance in one of the cultivars (Twilight) that lacked any *sbm* genes. In consequence, van Leur et al. [10] suggested field pea cultivars which are heterogeneous for *sbm1* gene resistance will need to be purified before being employed as parents for field pea breeding program crosses. They also emphasized that, although cultivars with resistance gene *sbm2* may appear to be PSbMV-resistant, their use might select for pathotypes P-1 and P-4. This is because P-3 pathotype is now known to be the most prevalent pathotype in Australia [10]. Furthermore, Congdon et al. concluded that, to minimize the chances of *sbm1* gene resistance being overcome, a greater focus on PSbMV management efforts by pea breeders, extension workers and growers was needed [9].

In a review which included Australian co-authors published in 2014, Makkouk et al. emphasized that PSbMV epidemics are economically important not only in field pea but also in lentil [26]. Although not yet considered important in lentil in Australia, widespread PSbMV infection of lentil crops causes major losses in the center of origin of both field pea and lentil in south-west Asia (the fertile crescent region), east Africa (Ethiopia) and the Indian subcontinent (Pakistan). In addition to the partial PSbMV resistance (including resistance to seed transmission) found in field pea and lentil, and the *sbm* PSbMV resistance genes present in field pea, there is also a *sbm* PSbMV resistance gene in lentil [26], which could be used in Australia to breed lentil cultivars with PSbMV resistance. Moreover, the partial PSbMV resistance already identified in some field pea and lentil lines in Australia [4,5] could be used by field pea breeders as the genetic background for *sbm* resistance, or alone as a source of resistance.

No attempts have been made in Australia to determine whether genetic engineering with viral gene constructs can introduce effective PSbMV resistance into field pea or other cool-season pulses. However, Australian studies found this approach proved ineffective at introducing stable virus resistance with CMV and BYMV in lupin [65]. Genetic modification by RNA silencing and genome editing seem more likely to be effective methods to employ for this purpose [66,67]. Linking speed breeding [68,69,70,71] with these approaches would facilitate efficient introduction of PSbMV resistance into field pea, lentil or other cool-season pulses.

## 8. Phytosanitary, Cultural and Chemical Control Measures

**Figure 6:**
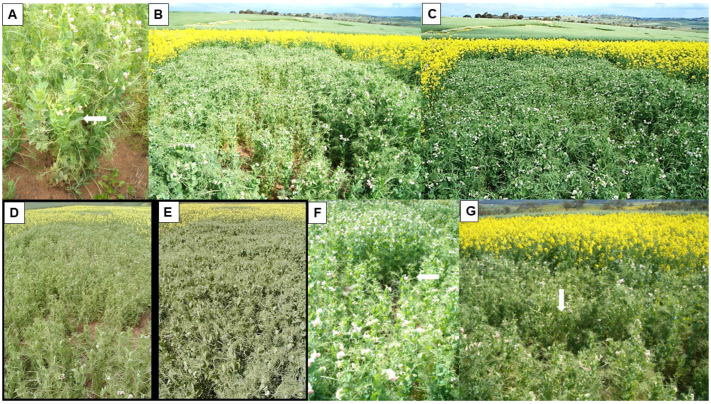
Field experiment investigating phytosanitary control by establishing threshold levels of pea seed-borne mosaic virus (PSbMV) infection in field pea (*Pisum sativum*) cv. Kaspa seed stocks (at Avondale in 2005). Plots separated from each other by 10 m-wide non-host canola (*Brassica napus*) buffers (yellow flowers). (**A**) Infector plant of faba bean cv. Fiord (*Vicia faba*) growing up after being transplanted into a plot of field pea to simulate a seed-infected field pea plant infection focus (white arrow points to faba bean infector plant). (**B**,**C**) Comparison of plots originally transplanted with sufficient faba bean infector plants to simulate 2.2% seed-borne infection (**B**) or sowing with 0.3% infected seed (**C**). This resulted in sufficient natural PSbMV spread to elicit foliage pallor, plant stunting and an uneven canopy, especially along the middle of the plot (**B**) versus vigorous normal-looking growth and a uniform canopy (**C**). (**D**,**E**) Comparison of plots originally sown with 6.5% (**D**) or 0.3% (**E**) infected field pea seed resulting in widespread natural PSbMV spread, which was sufficient to cause foliage pallor and an uneven plot canopy with depressions based on primary infection foci in (**D**), but only vigorous growth with a uniform canopy in (**E**). (**F**) Central circular central depression in growth containing pale stunted field pea plants (white arrow) based on plant stunting caused by natural spread of infection from a single central primary infection focus. (**G**) Central elongated depression in growth containing pale stunted field pea plants (white arrow) based on plant stunting caused by natural spread of infection from several primary infection foci. (**C**,**D**) Images modified from Coutts et al. [6].

In 2005–2006, three large-scale, replicated field experiments were undertaken with field pea cv. Kaspa at two sites within the south-western WA grainbelt (Figure 6A–G) [6]. In 2005, there were two experiments, one at Avondale and the other at Badgingarra (field experiments 1 and 2 respectively), whereas the single experiment in 2006 was at Avondale (field experiment 3). Both sites are in the grainbelt’s high rainfall zone (Table 2). Their purpose was to (i) quantify the seed yield losses and degree of infection in harvested field pea seeds likely to occur after seed stocks with different PSbMV infection levels are sown by farmers; and (ii) establish threshold levels of infection suitable for field pea seed stocks intended for sowing within regions with different risks of virus spread. Each plot was surrounded by a non-host species buffer of canola (*Brassica napus*) 10 m in width. The primary infection foci in the plots were either seed-infected pea plants, or PSbMV pathotype P-2 isolate W1-infected faba bean transplants that simulated them (Figure 6A). Naturally occurring, migrant winged aphid vectors spread PSbMV from infected to healthy plants. The actual or simulated seed transmission rates in sown seed were 0.3 to 6.5% (2005) or 0.1 to 8% (2006), and the extent of PSbMV spread depended upon the extent of primary inoculum (i.e., numbers of seed-infected/or infected transplanted plants) present, and climatic conditions that hastened or delayed the time of the first arrival of migrant aphid vectors. Since a healthy cv. Kaspa seed stock was unavailable, plots sown with 0.3% (2005) or 0.1% (2006) infected seed served as controls. There were two sets of these control plots in each experiment: one left unsprayed and the other sprayed four times with a combination of α-cypermethrin and imidacloprid insecticides during the growing season (Table 2). The climatic conditions favored early or late aphid arrival in 2005 and 2006, respectively. Figure 2B–D show details of the disease symptom effects of PSbMV spread from infection foci to nearby plants within these plots, and Figure 6B–G show the disease symptom effects of PSbMV spread upon overall plot appearance (at Avondale in 2005). When compared with the yields of plots sown with 0.3% infected seed without insecticide application, seed yields in plots sown with 6.5% (in 2005) or 8% (in 2006) infected seed were decreased by 18% at Avondale and 25% at Badgingarra in 2005, and 13% at Avondale in 2006. This was explained by final PSbMV incidence reaching 97% at Avondale and 98% at Badgingarra in 2005, but only 36% at Avondale in 2006 (Table 2). When 2% infected seed was sown in 2005, seed yield losses were 21% (at Avondale) and 15% (at Badgingarra), which occurred after final incidence reached 69% (at Avondale) and 76% (at Badgingarra). When 1% infected seed was sown in 2005, seed yield losses were significantly reduced only at one of the two sites (Badgingarra), where a 13% loss resulted from 48% PSbMV infection. By contrast, when 1 to 4% infected seed was sown in 2006 (at Avondale), there were no significant yield losses (Table 2). Plotting the relationships between % final incidence and yield for 2005 revealed a yield decline of 7.7 to 8.2 kg/ha for each 1% increase in PSbMV incidence. Seed-borne PSbMV infection after harvest was 1 to 17% in 2005 but only 0.2 to 2% in 2006. Thus, the extent of the PSbMV spread that occurred determined the magnitude of both seed yield losses and harvested seed infection. Based on these findings and those of Coutts et al. [5], from testing seed samples taken from commercial seed stocks intended for sowing, a <0.5% seed infection threshold was chosen as a phytosanitary control measure for seed stocks intended for sowing for food production in high-risk zones. Should field pea seed stocks with <0.5% seed infection be unavailable locally, high-risk zone farmers can source <0.5% infected seed for their sowings from low-risk zones. However, sowing 0.3% infected seed from crops with insecticide applied in high-risk situations still resulted in 1 to 0.2% seed infection. Therefore, retaining a <0.1% seed infection threshold was recommended for high-risk zone field pea crops grown entirely for seed needed for sowing in the following year [6].

In a French study published in 1988, in which seeds harvested from field pea plants sap inoculated with PSbMV were sown, planting small seeds (<6.5 mm sieve fraction) resulted in higher rates of seed-to-seedling virus transmission than planting larger seeds (>6.5 mm sieve fraction) [72]. As mentioned above (Section 4), SA studies with sap-inoculated field pea plants by Ligat et al. [14] had contradictory results. They found seed-to-seedling transmission rates to be lowest (27–40%) with the smallest seed fraction and highest (82–91%) with the largest. However, germination was also higher with the largest seed fractions. Research in WA published in 2017 examined the possibility of using seed-lot fractionation as an additional phytosanitary control measure to diminish PSbMV infection levels in commercial semi-leafless field pea seed stocks intended for sowing [73]. By passing seed through sieves differing in mesh sizes, six seed lots of field pea cvs Kaspa (4) and Twilight (2) were each sieved into three fractions (<6.0, 6.0 to 6.5 and >6.5 mm). When the sieved seeds were sown and the seedlings produced tested for PSbMV infection, % PSbMV seed-to-seedling transmission rates were significantly smaller in the >6.5 mm than in the <6.5 mm fraction. In one instance, this was also the case between the 6.5 mm and 6.0 to 6.5 mm seed fractions. Thus, applying differential sieving to remove smaller sized seeds could be a useful approach towards decreasing PSbMV infection levels below the 0.5% seed transmission risk threshold for sowing. Seed fractionation resulting in a higher proportion of small seeds could also provide an indication of high PSbMV seed infection levels too high to be suitable for sowing in high PSbMV risk zones.

In 2007, field pea cv. Kaspa seed with 0.5% or 8.2% PSbMV infection was sown in replicated field experiments at two different WA sites (Table 2) [6]. The purpose of these experiments was to explore using two cultural control measures (high seeding rate and straw mulch application) and a single chemical (regular insecticide application) control measure to reduce PSbMV spread and infection of harvested seed below % infection threshold levels in high value crops grown entirely for seed needed for sowing. The sites used were near Esperance and Merredin in the grainbelt’s high and low rainfall zones, respectively. Both experiments compared the spread of PSbMV infection in plots sown at a high seeding rate (200 kg/ha) in presence of straw mulch added at 2 t/ha and regular insecticide applications (a mixture of α-cypermethrin and imidacloprid), with the spread in plots sown at the standard seeding rate (100 kg/ha) without straw mulch being present or any insecticide applications. These two treatment combinations were repeated for plots sown with 0.5% or 8.2% infected seed. In addition, plots in which PSbMV-resistant cv. Yarrum seed was sown at the low seeding rate without straw mulch or insecticide applications were also present. Each plot was surrounded by a non-host species canola buffer 10 m in width. In field experiment 4, which was sown at the low-PSbMV-risk site (near Merredin), plant growth was stunted due to drought and PSbMV spread slowly. The outcome was that the final PSbMV incidence of 54% in the cv. Kaspa plots sown with 8.2% infected seed at the standard seeding rate without straw mulch or insecticide was significantly larger than in the other plots sown with this cultivar (32% for 8.2% infected seed and 3–4% for 0.5% infected seed) (Table 2). For plots sown with 8.2% seed infection, PSbMV infection levels in harvested seed were 10% (plots with standard seeding rate minus mulch and insecticide) and 4% (plots with high seeding rate plus mulch and insecticide), whereas, for plots sown with 0.5% infected seed, the corresponding figures were 0.8% (standard seeding rate minus mulch and insecticide) and 0.3% (high seeding rate plus mulch and insecticide) for harvested seed infection. In field experiment 5, which was sown at the high-risk site (near Esperance), PSbMV spread faster. At 98 days from sowing plots with 8.2% infected seed, there was significantly greater PSbMV spread in the plots sown at the standard seeding rate without straw or insecticide (98% incidence) than in the plots sown at high seeding rate with straw and insecticide (79% incidence) (Table 2). In addition, in plots sown with 0.5% infected seed at 98 days, there was also significantly greater PSbMV spread in plots sown at the standard seeding rate without straw or insecticide (83% incidence) than in the plots sown at high seeding rate with straw and insecticide (50% incidence). However, by 111 days from sowing, this experiment had become overwhelmed by PSbMV spread, with 96–100% incidences being reached regardless of initial seed infection level as well as presence or absence of straw mulch or insecticide. For plots sown with 8.2% infected seed, PSbMV infection levels in harvested seed were 36% (standard seeding rate minus mulch and insecticide) and 23% (high seeding rate plus mulch and insecticide), whereas, for plots sown with 0.5% infected seed, the corresponding figures were 25% and 15% for harvested seed infection (Table 2). No PSbMV was ever detected in the cv. Yarrum plots in either experiment. Thus, in only one instance in these two experiments (when the high seeding rate, mulch and insecticide were used at the low-risk site) was the output PSbMV seed transmission rate smaller (0.3%) than the input seed transmission rate (0.5%). This finding indicates that, unless a seed stock with <0.5% is sown, combining high seeding rate, straw mulch and insecticide application may not be adequate to diminish PSbMV infection below the <0.5% infection threshold for sowing and therefore not be a reliable measure when growing field pea crops for seed multiplication.

The key findings described above concerning management by phytosanitary and cultural control measures were used to develop an IDM strategy for PSbMV in field pea. This IDM strategy is described below in Section 10 along with (i) recommendations for further research on phytosanitary and cultural control measures in both eastern Australia and WA, and (ii) interim additions to the PSbMV IDM derived from Australian studies with other seed-borne pulse viruses.

## 9. Epidemic Drivers and Forecasting

**Figure 7:**
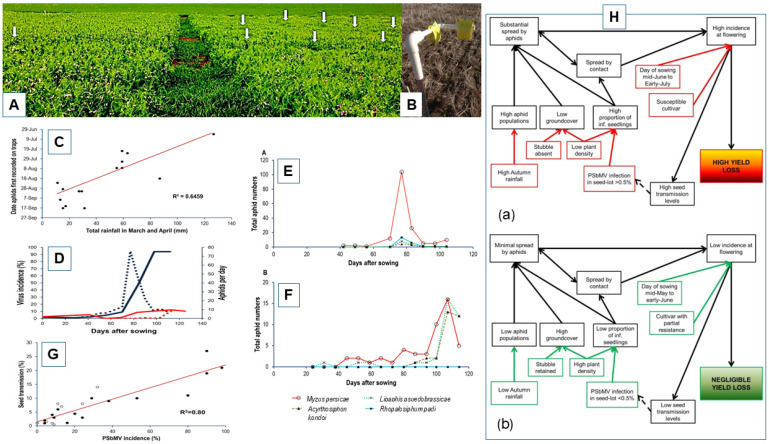
Pea seed-borne mosaic virus (PSbMV) field pea pathosystem epidemic drivers. (**A**) Field expression of PSbMV disease in field pea showing as canopy depressions at flowering time (white arrows) arising from spread from seed-infected plants acting as primary infection foci. Block sown with high seed infection (Kaspa, 13%, on right) developed deeper and more extensive canopy depressions than block sown with lower seed infection (Twilight, 2%, on left), reflecting a greater internal virus source driving earlier and faster within-crop spread. (**B**) Two double-sided yellow sticky traps set orthogonally to each other were used to measure migrant aphid activity from four directions. (**C**) Relationship between March and April (i.e., autumn) rainfall and timing of first migrant aphid arrival based on yellow sticky trap data. Higher early-autumn rainfall caused earlier arrival of winged aphids (early July–early August), whereas lower early-autumn rainfall delayed aphid arrival until later (August–September), strongly influencing the window of crop vulnerability to PSbMV spread. (**D**) Contrasting scenarios for aphid numbers (dotted lines) and virus spread (solid lines) when 2% PSbMV-infected cv. Kaspa seed was sown in different years, in 2011 at Grass Patch (red color) and in 2014 at Bolgart (black color). Late aphid arrival in 2011 resulted in minimal virus spread whereas early aphid arrival resulted in all plants being infected. This comparison demonstrates that pre-sowing rainfall modulates whether a given seed infection level will be amplified into a damaging epidemic. (**E**,**F**). Seasonal dynamics of migrant aphid species caught in green tile traps at representative sites in 2014 (Bolgart) (**E**) and 2015 (Muresk) (**F**), showing that aphid species such as *Myzus persicae*, *Lipaphis pseudobrassicae*, *Acyrthosiphon kondoi* and *Rhopalosiphum padi* dominated flights into field pea data-collection blocks. (**G**) Seed transmission of PSbMV in harvested seed as a function of crop incidence at flowering, showing a strong positive relationship independent of cultivar (Kaspa) (●) or Twilight (○). (**H**) Conceptual epidemiology model for the PSbMV field pea pathosystem in a Mediterranean-type climate; (**a**) High risk, (**b**) Low risk; Red lines (high risk), green lines (low risk); dotted line arrows indicate feedback from transmission rates in harvested seed to levels in seed stocks planted in the following growing season. This framework shows how pre-sowing rainfall influencing migrant aphid abundance and PSbMV infection in seed sown, combine to determine the extent of PSbMV spread in field pea crops up until flowering time; this in turn determines the amount of yield loss and virus transmission to harvested seed. (**A**–**C**) and (**E**–**H**) modified from Congdon et al. [74].

Section 8 above described how WA grainbelt rainfall zones and climatic conditions before the start of the growing season (especially rainfall) determine when aphid vectors first arrive in crops, the extent of PSbMV spread that results from seed-infected plant infection foci and subsequent secondary infection sources within the crop, and the magnitude of resulting losses in seed yield and infection of harvested seed. This information came from field experiments done in 2005–2007 in which field pea seed stocks with different levels of PSbMV infection were sown or simulated at five sites in the WA grainbelt [6]. In 2010–2015, further information on the factors driving PSbMV epidemics under the autumn to spring growing conditions in this region was obtained from 23 square (60 × 60 m) data-collection blocks sown at 2–5 sites each year at sites in different regions of the grainbelt [74]. Their sowing dates were between 25 May and 22 June each year, the field pea cultivars sown were Kaspa every year and Twilight in 2012–2015 (2 blocks/site), and the seed sown had 0.1–13% PSbMV infection (Figure 7A). The information collected included daily growing season rainfall data from the nearest Bureau of Meteorology Weather Station, time of aphid arrival in each block, numbers of winged aphid migrants present, PSbMV incidence throughout the growing season, and PSbMV seed transmission rates to seedlings grown from harvested seed. Yellow sticky traps changed on a weekly basis monitored overall aphid movement (Figure 7B), and, in 2014 and 2015, green tile traps enabled monitoring of individual aphid species landing in the blocks, specifically *M. persicae*, *R. padi*, *A. kondoi* and *L. pseudobrassicae*, the last of which is not known to be a PSbMV vector. In addition, a central 20 × 20 m square within each block was used to monitor aphid colonization but none occurred. Every 2 weeks, this square was also used to collect PSbMV incidence data by testing 100 randomly collected shoot samples for PSbMV. A 1000-seed sample harvested from each block was tested for PSbMV transmission to seedlings.

Aphid migrants first arrived at the blocks between 33 and 129 days after sowing based on yellow sticky trap data [74]. Higher early-autumn rainfall caused earlier arrival of winged aphids (early July–early August), whereas lower early-autumn rainfall delayed aphid arrival until later (August–September), strongly influencing the window of crop vulnerability to PSbMV spread. Figure 7C shows a straight-line relationship between the extent of March and April rainfall and first migrant aphid arrival. A diverse range of epidemic scenarios resulted, depending upon first aphid arrival time. For example, these ranged from a high pre-sowing rainfall situation (125 mm of rain in March and April), with 13% infection in seed sown in which aphids arrived at 33 days from sowing and PSbMV spreading fast, reaching a final incidence of 100% and a harvested seed infection rate of 21% (cv. Kaspa, at Grass Patch in 2013), to a lower pre-sowing rainfall situation (31 mm), with 7% infection in seed sown in which aphids arrived 129 days from sowing and PSbMV spread reached a final incidence of 24% and a harvested seed infection rate of 3% (cv. Kaspa, at Wittenoom Hills in 2010). Another example was when 2% PSbMV-infected cv. Kaspa seed was sown at two sites in different years: 2011 (at Grass Patch) and 2014 (at Bolgart) (Figure 7D). In 2011, the low pre-sowing rainfall situation (26 mm of rain in March and April) resulted in late aphid arrival 120 days after sowing, resulting in only 9% final incidence and 4% seed infection, whereas, in 2014, the intermediate pre-sowing rainfall situation (58 mm rain in March and April) resulted in mid-season aphid arrival (50 days after sowing), resulting in a 94% final incidence and 10% seed infection. Figure 7E,F show the seasonal dynamics of migrant aphid species caught in green tile traps over both cultivars (Kaspa and Twilight) at representative sites in 2014 (E) and 2015 (F). Before flowering, the PSbMV incidence in each block depended upon two primary epidemic drivers: (i) the amount of PSbMV infection in sown seed, which determines the potency of the primary inoculum source for spread by aphids or contact transmission, and (ii) the magnitude of early-autumn to mid-autumn (March–April) rainfall that enables growth of background vegetation, which itself determines the extent of early aphid population build-up and the time when aphid migrants first reach field pea crops. Sowing date was identified as an important secondary epidemic driver that determined which plant growth stage would be exposed to aphid migrants. Also, a critical factor determining % PSbMV harvested seed infection was % PSbMV incidence at flowering time (Figure 7G) [74]. Figure 7H provides a PSbMV field pea pathosystem conceptual epidemiology model for regions with Mediterranean-type climates. In brief, it explains how pre-sowing autumn rainfall driving migrant aphid abundance and PSbMV seed infection both determine the amount of PSbMV spread up until flowering time, which then dictates yield loss extent and harvested seed virus transmission to seedlings [74].

**Figure 8:**
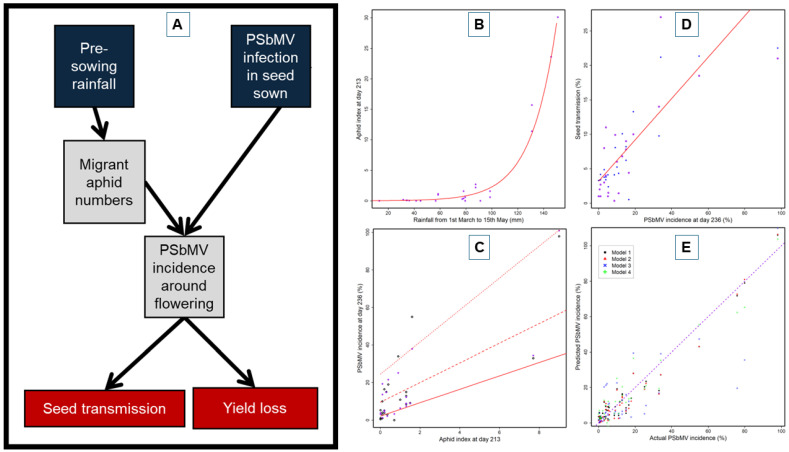
Forecasting pea seed-borne mosaic virus (PSbMV) epidemics in field pea crops in a Mediterranean-type climate. (**A**) Simplified conceptual model showing main parameters (black for inputs, grey for within growing season and red for outputs) and relationships involved in the PSbMV field pea forecasting model. General relationships were established in advance from epidemiological data collected in the south-west Australian grainbelt, which has a Mediterranean-type climate. The parameter periods and dates used were established through a model calibration process. (**B**) Exponential relationship between pre-sowing rainfall (1 March to 15 May) and aphid abundance at day 213, showing that wetter autumns generate larger aphid populations capable of driving early and severe PSbMV epidemics. (**C**) Relationship between migrant aphid abundance (aphid index at day 213) and PSbMV crop incidence at day 236 (late August), demonstrating that virus spread is strongly dependent upon aphid activity and infection incidence in sown seed. Lines represent model predictions (purple spot = predicted and black spot = actual) for different levels of seed infection (SI) [SI level in % =13 (dotted line), =5 (dashed line) and =1 (solid line)], illustrating the strong aphid × SI interaction that underpins PSbMV epidemic development. (**D**) Relationship between PSbMV crop incidence at day 236 and virus transmission from harvested seed-to-seedling (blue spot = predicted, purple spot = actual), indicating that higher virus incidence before flowering leads to a greater magnitude of transmission into the next generation of seed, thereby amplifying epidemic risk in subsequent growing seasons. (**E**) Validation of four alternative empirical forecasting models comparing predicted versus actual PSbMV incidence at day 236, with model 2 providing the highest predictive accuracy (R^2^ ≈ 0.94; mean absolute error ≈ 4.6%), enabling robust pre-sowing risk forecasts for decision support in field pea grown in Mediterranean-type climates. (**A**–**E**) Modified from Congdon et al. [75].

A combination of epidemiological information from the data-collection blocks of Congdon et al. [74] described in the previous paragraph, and the five field experiments of Coutts et al. [6] described above in Section 8, was used to develop, calibrate and validate a model designed to predict PSbMV epidemics in field pea crops growing in Mediterranean-type climatic conditions [75]. After exploring the possible use of a mechanistic model like that of Thackray et al. [76] for CMV in narrow-leafed lupin, the lack of sufficient information about background soil moisture, vegetation and aphid numbers meant that an empirical model was more suitable with PSbMV infection of field pea [75]. This model’s aim was to predict seed yield losses in PSbMV-infected crops by forecasting the incidence of PSbMV infection at a growing season phase found to be crucial for this purpose. An index of aphid abundance in early-August was calculated based on pre-growing season rainfall data. When combined with data on the level of infection with PSbMV in sown seed, this index was used to forecast PSbMV incidence in field pea crops. Sowing date and the predicted incidence of PSbMV crop infection in early-August were then used to predict the PSbMV seed-to-seedling transmission rate for harvested seed (Figure 8A). Figure 8B shows the relationship between pre-sowing autumn rainfall (1 March to 15 May) and aphid umbers at day 213. This reveals how drier autumns generate smaller aphid populations incapable of driving early and severe PSbMV epidemics, whereas wetter autumns generate larger aphid populations capable of doing this. Figure 8C shows the relationship between migrant aphid numbers (aphid index at day 213) and PSbMV incidence at day 236 (late August), clarifying how PSbMV spread depends upon aphid activity and infection incidence in sown seed. Figure 8D shows the relationship between PSbMV incidence at day 236 and PSbMV seed-to-seedling transmission rates in harvested seed, revealing that greater PSbMV incidences before flowering result in increased PSbMV transmission in the next generation of seed, thereby enhancing epidemic risk in later growing seasons. Figure 8E compares predicted versus existing PSbMV incidence at day 236 from four alternative empirical forecasting models. Model 2 delivered the greatest predictive accuracy, providing the most accurate pre-sowing risk forecasts.

Since the model predicted PSbMV crop incidence accurately when pre-sowing time data were included, it was suitable for forecasting end-user yield and income losses. To allow sufficient time for control recommendation implementation when crops are sown, forecasts for the period prior to sowing were necessary. Such control measures included sowing PSbMV-resistant cultivars (host resistance) or seed stocks of susceptible cultivars with below <0.5% threshold PSbMV infection levels (phytosanitary) and deploying cultural control approaches likely to help (Section 8 above). The model’s disease risk forecast allowed for both the prevailing economic circumstances encountered in field pea production and the predicted % yield loss caused by PSbMV infection. It provided end users with PSbMV management recommendations that were location-specific and deliverable through SMS alerts linked to web support information about PSbMV and how to control it in field pea crops. It also improved end-user cost effectiveness by enabling their avoidance of control measure deployment when this was unnecessary [75].

## 10. Integrated Disease Management

Control measures act by minimizing either virus infection sources or rates of virus spread [77,78,79,80,81,82]. For each virus and crop combination, comprehensive knowledge of its epidemiology and the effectiveness and mode of action of each individual control measure is needed before an effective IDM approach can be devised. When a suitable combination of phytosanitary, cultural and host resistance is deployed, the likelihood of major yield losses occurring is minimized. Understanding the mode of action of each control measure requires knowledge of whether its selectivity is general or specific, and whether it targets internal or external virus sources, or whether virus spread occurs earlier or later within the crop. IDM effectiveness is maximized by deploying a mix of different types of control measures that act in different ways, not only including ones with high and low selectivity, but also ones that ensure both internal and external virus sources are targeted, and both early and late phases of virus spread within the crop are addressed [81,82].

The three components of the current IDM strategy for the PSbMV/field pea pathosystem backed by comprehensive Australian research (see Section 7 and Section 8 above), are:

- Only sow field pea seed stocks with below the <0.5% threshold level of PSbMV seed infection in grainbelt zones with a high level of PSbMV epidemic risk (phytosanitary).
- Sieve out smaller seeds to reduce the virus content of PSbMV-infected seed stocks intended for sowing (phytosanitary).
- Sow field pea cultivars with resistance; either single gene PSbMV resistance, especially ones with the *sbm1* gene, and/or partial PSbMV resistance (host resistance).

In eastern Australia, field experiments on phytosanitary control like those described above for WA in Section 8 are urgently required to establish safe threshold levels of seed-borne PSbMV infection for sowing in grainbelt regions with different levels of virus infection risk. Moreover, in all Australian states where field pea is an important crop, field experiment studies are required involving cultural control measures like those conducted previously with CMV and BYMV in lupins in WA and reviewed recently in Volumes 1 and 2 [1,2]. The two 2007 cultural control field experiments described above in Section 8 only represent an initial step towards achieving this objective, so further experiments investigating the effectiveness of cultural control measures against PSbMV spread in field pea crops are required. These need to be aimed at reducing: (i) seed-infected plant survival, thereby diminishing the primary infection source dispersed at random throughout the crop; (ii) secondary infected plant survival, thereby delaying expansion of the secondarily infected plant source, which clusters initially around individual primary infection sources but subsequently becomes widely dispersed within crops; (iii) aphid vector landings within the crop, thereby diminishing virus acquisition from primarily or secondarily infected plants followed by its spread to healthy plants within the crop; and (iv) initial aphid vector colonization of vulnerable young plants within the early phase of crop growth. Sowing at greater depth can reduce the survival or delay the emergence of seed-infected seedlings to a greater extent than vigorous healthy seedlings. In addition, sowing at high seedling rates increases within row plant density, which helps diminish virus-infected seedling survival due to competition with neighboring, more vigorous healthy plants. It can also help shade out early secondarily infected plants. However, this effect diminishes once aphid vectors arrive in larger numbers and increase the process of spreading infection to nearby healthy seedlings. Diminished aphid landing rates reduce secondary virus spread by aphids. The presence of bare earth between rows stimulates aphid landings, but cereal stubble on the soil surface between rows prior to canopy closure has the opposite effect. As narrow row spacing promotes early canopy closure, it decreases aphid vector landing rates once this closure is in place. The high plant density within rows that results from sowing at high seeding rates also promotes earlier canopy closure, so also reduces aphid landing rates. In addition, altering the sowing date to avoid young vulnerable plants being present at peak aphid vector population times, sowing early maturing cultivars to avoid peak aphid vector population times at the end of the growing season, and avoiding sowing in fields next to other crops likely to act as external virus sources, are other cultural control measures that deserve attention in the future [1,2,81,83,84]. If contact transmission from damage to foliage by wind currents or agricultural machinery passing through them enlarges primary infection foci within young crops, two cultural control options likely to help are (i) planting wind breaks consisting of closely spaced tall trees around field perimeters like those used in New Zealand’s Canterbury plains region [85]; and (ii) avoiding contact between agricultural machinery and young plants. In addition, spraying mineral oils onto crop foliage constitutes a chemical control method not involving insecticide application that has proved effective in limiting spread by aphid vectors of other potyviruses [86], which is likely to help in reducing PSbMV spread, particularly in high-value field pea crops being grown for seed multiplication.

For virus–host pathosystems lacking research on specific virus control measures that have already proven effective when studied with closely related pathosystems, the value of including them within interim IDM strategies prior to their experimental validation is well understood [80,81,87]. Therefore, in the interim period prior to completion of the studies suggested in the previous paragraph, based on the findings of previous research with Australian CMV and BYMV/narrow-leafed lupin pathosystems [1,2], inclusion of the following measures within the PSbMV field pea IDM would likely prove worthwhile:

- Sow seeds at high seeding rates to generate high plant densities within rows that shade out seed-infected and early current-season infected seedlings, thereby minimizing the early internal virus infection source for subsequent PSbMV spread by aphid vectors (cultural).
- Maximize stubble groundcover prior to canopy closure by soil cultivation using minimum tillage, thereby reducing aphid vector landing rates and consequent PSbMV spread (cultural).
- Combine sowing seeds at narrow row spacing with sowing at high seeding rates in presence of retained stubble to diminish aphid vector landing rates prior to canopy formation (cultural).
- Sow early-maturing field pea cultivars to minimize the extent of PSbMV spread by vector aphids and harvested seed infection late in the growing season (cultural).
- Alter the sowing date to avoid exposure of vulnerable young plants to aphid vector population peaks (cultural).
- Ensure isolation from neighboring field pea or other pulse crops to avoid any ingress of PSbMV from vector aphids flying from them (phytosanitary).

## 11. Conclusions

Over the almost six decades since PSbMV’s presence in the Australian continent was first reported in 1978, the many research findings accumulated involving this virus and the seed-borne diseases it causes in Australia’s cool-season pulse crops have greatly increased knowledge about the virus disease threat it poses to the country’s large and profitable domestic and export pulse industry. Historically, most of the research on PSbMV was done in SA (published in 1991–2007), WA (published in 2001–2017), and NSW and VIC (usually published jointly in 2005–2025). In brief, these research activities included: characterising its symptomatology in foliage and seeds; improving sample testing procedures; aphid, seed and contact transmission; pathotyping isolates and nt sequence phylogenetics; phenotyping and host resistance studies; evaluating potential phytosanitary, cultural and chemical control measures; devising an IDM strategy; identifying epidemic drivers; and developing a forecasting model and decision support system. The most widely grown cool-season pulse crop in Australia (lupin) proved to be a PSbMV non-host, but field pea, lentil, faba bean and chickpea, and all nine minor cool-season pulse species grown in the continent, became infected. The PSbMV/field pea pathosystem is most studied due not only to PSbMV’s common occurrence in commercial field pea crops and seed stocks, but also to its detrimental impact on seed yield and quality demonstrated in both glasshouse and field experiments. Although it also causes serious diseases in lentil, faba bean and chickpea, and was seed-borne in all three of them, PSbMV infection occurred less often in the crops, plots and seed stocks of these three pulse species.

The principal achievements of past Australian research on PSbMV infection of cool-season pulses include:

- Diverse foliage symptom types that develop in infected field pea, lentil, faba bean and chickpea, and nine minor pulse species (narbon bean, vetches and *Lathyrus* species), were characterized.
- Diverse seed coat symptom types were caused by PSbMV, and occurred in seeds of field pea, faba bean, lentil and chickpea, and six minor pulse species (vetches and *Lathyrus* species).
- None of the nine lupin species tested became PSbMV-infected (narrow-leafed, white, yellow and pearl lupin and five rough-seeded lupin species).
- The cool-season pulse-colonizing aphid species *M. persicae*, *Aphis craccivora*, *A. kondoi* and *A. pisum,* and non-colonizing species *R. padi*, act as PSbMV vectors, the most important being *M. persicae*, *A. kondoi* and *R. padi*.
- Wind-mediated contact transmission of PSbMV, which would expand initial crop infection foci in the field prior to aphid vector arrival, was demonstrated under conditions that simulated sand and dust particle blasting of field pea foliage due to wind currents.
- Seed-to-seedling transmission of PSbMV was recorded for field pea seed lots from the field (up to 47%), lentil (up to 6%), faba bean (up to 2%), chickpea (0.1%), and the minor pulses common vetch (0.3%), purple vetch (0.1%), grass pea (1%), dwarf chickling (0.4%), *L. clymenum* (5%) and *L. ochrus* (0.7%).
- Sowing PSbMV-infected seed stocks resulted in primary infection foci consisting of seed-infected plants dispersed at random throughout field pea crops. These infection foci constituted the principal source for secondary virus spread by aphid vectors or, potentially, by wind-mediated contact transmission.
- Only 4/19 pasture or forage legume species tested became PSbMV-infected. Amongst these, <5% of plants/species were infected (ranked as resistance), showing only asymptomatic infection. Therefore, they are unlikely to act as significant alternative hosts for PSbMV spread to pulse crops.
- When both commercial crops and seed stocks, and experimental plots and their seed lots, were surveyed between 1984 and 2015 throughout southern Australia, PSbMV infection occurred commonly in field pea but far less often in faba bean, lentil and chickpea.
- PSbMV pathotypes P-1, P-2, P-3 and P-4 were differentiated by their interactions with field pea resistance genes *sbm-1* and *sbm-2*, P-3 being the commonest and P-2 the least common. Different PSbMV isolates varied in virulence regardless of pathotype, with severe P-4 and P-2 isolates from field pea eliciting an 84% seed yield loss and 31% seed transmission rate in field pea, and a 96% yield loss and 6% seed transmission rate in lentil, respectively.
- In PSbMV field studies, field pea, faba bean and lentil genotypes ranked from highly susceptible to resistant, five field pea genotypes being highly resistant. Chickpea genotypes were susceptible or moderately resistant. Narbon bean and dwarf chickling genotypes were highly susceptible, susceptible or moderately resistant, whereas *L. ochrus*, common vetch, purple vetch and bitter vetch genotypes were moderately resistant or resistant. Grass pea genotypes were resistant or highly resistant. Foliage sensitivity rankings varied from high in some faba bean, Kabuli chickpea, narbon bean and dwarf chickling genotypes, to moderately sensitive or tolerant with field pea, and mostly tolerant with lentil.
- When many field pea lines were evaluated for PSbMV resistance in NSW field experiments, only cvs Yarrum, Maki and Walana, and breeding lines OZP805 (later released as cv. Wharton) and G-1000, carried resistance gene *sbm1*. Also, partial PSbMV resistance was found in cvs Glenroy and Soupa, and cvs Cressy Blue, Boreen and Mukta had resistance to PSbMV seed transmission.
- When field pea seed stocks with actual or simulated PSbMV seed transmission rates of 0.3 to 6.5% or 0.1 to 8% were sown in replicated field experiments, the extent of virus spread determined the magnitude of seed yield losses and harvested seed infection. Based on the outcomes of these experiments and testing 1000-seed samples from commercial seed stocks, a <0.5% seed infection threshold was chosen for seed stocks intended for sowing in high-PSbMV-risk zones.
- When sieved field pea seeds were sown, % PSbMV seed transmission rates were smaller in >6.5 mm than <6.5 mm fractions, so removing smaller seeds can be used to help diminish PSbMV infection levels below the 0.5% risk threshold for sowing seed stocks.
- When two field experiments compared PSbMV spread in plots sown with 0.5% or 8.2% infected field pea seed, sown at a high seeding rate in presence of straw mulch and regular insecticide applications with the PSbMV spread in similar plots sown at standard seeding rate, without straw mulch or insecticide applications, only once, when 0.5% infected seed, high seeding rate, mulch and insecticide were used, was the output seed transmission rate smaller than the input seed transmission rate.
- The IDM strategy devised for the PSbMV field pea pathosystem consists of sowing seed stocks with below the <0.5% threshold level of PSbMV seed infection, sieving infected seed stocks to remove smaller seeds and sowing cultivars with resistance gene *sbm1* and/or partial resistance. Adding cultural control measures that proved effective against CMV and BYMV spread in narrow-leafed lupin is suggested, pending similar field experimentation with PSbMV in field pea.
- Field studies revealed that PSbMV incidence in field pea crops, and magnitude of yield losses and harvested seed infection, depend mainly upon two epidemic drivers: (i) amount of infection in sown seed, which determines primary inoculum source size for spread by aphids or contact transmission; and (ii) magnitude of early-to-mid-autumn rainfall, which supports background vegetation growth that determines early aphid population build-up and time when aphid migrants first reach crops. Secondary epidemic drivers include (i) sowing date, as this determines which plant growth stage will be exposed to aphid migrants and wind-mediated contact transmission localized around seed- or early-infected plants; and (ii) PSbMV incidence at flowering time determines % PSbMV seed infection.
- An empirical forecasting model was developed for the PSbMV field pea pathosystem. Pre-growing season rainfall was used to calculate an aphid vector abundance index. This index and the amount of infection in sown seed were used to forecast PSbMV crop incidence. Sowing date and predicted incidence were used to predict PSbMV transmission in seed. Predicted yield losses and economic costs were used to develop a decision support system for use by agricultural advisers and farmers. To allow sufficient time for control measures to be deployed when crops are sown, forecasts were provided for the period before sowing time.

Further research activity is required, especially with field pea, and in due course may also be needed with lentil. Future research priorities and research gaps include:

- More information is needed on the biological characteristics of diverse PSbMV strains found throughout Australia, especially about their aphid vector and seed transmissibility, virulence in field pea and lentil, and potential to break down resistance genes such as *sbm1* in field pea and *sbm* in lentil.
- Further PSbMV genomic sequences from different cool-season pulse species and pulse-growing regions is required to provide a more comprehensive picture of the genetic diversity of this virus within Australia, and where PSbMV originated and spread from to reach this continent.
- More research activity is required to adapt the latest advances in virus testing procedures to ensure their suitability for use with PSbMV in cool-season pulses. The objective is to achieve not only greater accuracy and cost effectiveness when many samples need to be tested, but also to extend their use beyond plant health activities to PSbMV and aphid vector surveillance and management.
- In preparation for possible future breakdown of *sbm* resistance genes arising from the appearance of resistance-breaking PSbMV strains, the potential for employing the partial resistance to infection and resistance to seed transmission found in some field pea genotypes in Australia needs to be explored.
- Use of RNA silencing and genome editing is warranted as it has the potential to enhance PSbMV resistance in field pea breeding programs; employing speed breeding would facilitate the incorporation of this trait.
- Field experiments are needed to establish the extent to which wind-mediated contact is a significant contributor to PSbMV spread in commercial crops of field pea, and the use of remedial measures such as wind breaks is warranted in the worst wind-affected regions.
- Field experiments evaluating phytosanitary control measures like those described in Section 7 are required to establish safe threshold levels of % seed-borne PSbMV infection for sowing in eastern Australian grainbelt regions where there is a high risk of virus-induced losses in field pea crops.
- In all Australian regions where field pea is widely grown, field experiments evaluating potential cultural control measures like those discussed in Section 8 are needed to establish whether sowing at high seedling rates with narrow row spacing and straw mulch present reduces PSbMV spread due to shading out primary (i.e., seed-infected) or early secondary infection foci within rows, and reduces aphid landing rates by increasing groundcover before canopy closure or generating early canopy cover.
- Field experiments evaluating other potential cultural control measures mentioned in Section 8 are needed to establish whether altering sowing time or sowing early maturing cultivars can diminish exposure of vulnerable young plants to aphid vector population peaks or minimize PSbMV spread by vector aphids or late-growing season infection in harvested seed, respectively. Also, greater sowing depth might delay emergence and reduce survival of seed-infected seedlings.
- Field experiments are also needed to establish whether spraying mineral oils onto field pea crop foliage would be an effective chemical control method, not involving insecticide application, to limit PSbMV spread by aphid vectors.
- Field experiments designed to study the spatial and temporal dynamics of spread of PSbMV in field pea stand like those used to provide critical epidemiological information for the CMV/narrow-leafed lupin pathosystem, in which sowing virus-infected seed is the principal source of virus infection (Volume 1) and the BYMV/narrow-leafed lupin pathosystem (Volume 2) are also needed.
- The forecasting model devised to predict PSbMV crop incidence, % seed-to-seedling transmission in harvested seed, and end-user yield and income losses now needs to be adjusted to take account of recent changes in WA grainbelt climate and cultural practices. Also, its application elsewhere in Australia would require further adjustment and validation to take into account differences between states in climate and cultural practices. It requires ongoing support for its future deployment.

In addition, in other cool-season pulse crops apart from field pea, a watching brief needs to be maintained concerning both PSbMV’s occurrence in crops and seed stocks and the losses in yield and seed quality it is causing. This is especially so for lentil because, as mentioned above in Section 7, PSbMV is particularly damaging to this crop in some other parts of the world and virulent lentil-adapted PSbMV strains might become introduced inadvertently. Notably, within Australia, when a virulent PSbMV isolate from field pea infected lentil, it caused a yield loss of 96% and a 6% seed-to-seedling transmission rate in harvested seed.

Generic recommendations discussed in Volume 1 of this series for future Australian seed-borne virus disease research, which also apply to PSbMV, include (i) addressing difficulties in controlling virus disease epidemics originating from changing agricultural practices and an increasingly unstable climate; and (ii) adopting new technologies, e.g., combining precision agriculture with artificial intelligence and remote sensing to help establish overall vector and virus incidences and successfully target vector infestation and virus infection foci within crops with chemical control measures.

## Figures and Tables

**Table 1:** Principal foliage symptoms pea seed-borne mosaic virus infection causes in cool-season pulse species grown in Australia.

Pulses Grown		Leaf Symptoms	Shoot or Entire Plant Symptoms	Reference
Main crops				
Common name	*Botanical name*			
Chickpea	*Cicer arietinum*	Mild to moderate mosaic, mild to moderate chlorosis, downcurling	Apical tip necrosis, mild to moderate plant stunting	[4,5]
Faba bean	*Vicia faba*	Mild or moderate mosaic, deformation	Apical tip necrosis, mild to moderate plant stunting	[4,5]
Field pea	*Pisum sativum*	Mild to severe mosaic, mild to moderate deformation	Mild to severe plant stunting	[4,5]
Lentil	*Lens culinaris*	Mild to moderate mosaic, mild to moderate chlorosis, deformation	Apical tip necrosis, mild to severe plant stunting	[4,5]
Minor crops				
Grass pea	*Lathyrus sativus*	Mosaic, chlorosis, deformation	Apical tip necrosis, plant stunting	[4]
Dwarf chickling	*Lathyrus cicera*	Mosaic, chlorosis, deformation, necrotic spots and streaking	Apical tip necrosis, plant stunting, plant death	[4]
-	*Lathyrus clymenum*	Mosaic, downcurling	Necrotic stem streaking, apical tip necrosis, severe plant stunting	[4]
-	*Lathyrus ochrus*	Mosaic, chlorosis, deformation	Apical tip necrosis, plant stunting	[4]
Narbon bean	*Vicia narbonensis*	Mosaic, veinal necrosis, necrotic rings, spots, line patterns and streaking	Shoot tip distortion, apical tip necrosis, stunting	[4]
Purple vetch	*Vicia. benghalensis*	Mosaic, downcurling	Apical tip necrosis, plant stunting	[4]
Bitter vetch	*Vicia ervilia*	Mosaic	Apical tip necrosis, severe plant stunting	[4]
Common vetch	*Vicia sativa*	Mosaic, necrotic spots and streaking	Severe plant stunting	[4]

**Table 2:** Field experiments studying phytosanitary, cultural and chemical control measures against pea seed-borne mosaic virus (PSbMV) spread in field pea stands.

Experiment (Year)	Site (PSbMV Risk)	% PSbMV in Seed Sown	Treatments			Final % PSbMV Incidence	Seed Yield (t/ha)	% PSbMV in Harvested Seed
			Seeding rate (kg/ha)	Mulch	Insecticide			
Exp. 1 (2005)	Avondale (high)	0.3	100	-	+	4 a	2.92 a	0.2
		0.3	100	-	-	9 a	2.75 a	1
		0.7	100	-	-	38 b	2.61 a,b,c	2
		1.1	100	-	-	40 b	2.46 b,c	1
		1.4	100	-	-	61 c	2.45 b,c	2
		2.2	100	-	-	69 c	2.18 c	3
		6.5	100	-	-	97 d	2.26 c	11
*p=*						<0.001	0.03	
Exp. 2 (2005)	Badgingarra (high)	0.3	125	-	+	6 a	2.91 a	1
		0.3	125	-	-	9 a	2.91 a	3
		0.6	125	-	-	36 b	2.99 a	4
		0.9	125	-	-	48 b	2.65 a,b	5
		1.1	125	-	-	48 b	2.47 b,c	2
		2.7	125	-	-	76 c	2.44 b,c	6
		6.5	125	-	-	98 d	2.17 c	17
*p=*						<0.001	0.01	
Exp. 3 (2006)	Avondale (high)	0.1	100	-	+	4 a	2.59 a	0.2
		0.1	100	-		6 a	2.21 b	0
		0.25	100	-		6 a	2.41 a,b	0.1
		0.5	100	-		14 b	2.16 b,c	0.2
		1	100	-		15 b	2.33 b	0.3
		2	100	-		27 c	2.25 b	0.4
		4	100	-		28 c	2.14 b,c	0.8
		8	100	-		36 d	1.92 c	2
*p=*						<0.001	0.01	
Exp. 4 (2007)	Merredin (low)	8.2	100	-	-	54 d	0.08	10
		8.2	200	+	+	32 c	0.04	4
		0.5	100	-	-	4 b	0.08	0.8
		0.5	200	+	+	3 b	0.03	0.3
cv. Yarrum		0	100	-	-	0 a	0.07	-
*p=*						<0.001	ns	
Exp. 5 (2007)	Esperance (high)	8.2	100	-	-	98 d	1.83	36
		8.2	200	+	+	79 c	2.04	23
		0.5	100	-	-	83 c	1.74	25
		0.5	200	+	+	50 b	1.75	15
cv. Yarrum		0	100	-	-	0 a	2.03	-
*p=*						<0.001	ns	

Most important phytosanitary and cultural control field experiment findings. Full details available in Coutts et al. [6]. Field pea cultivar always PSbMV-susceptible cv. Kaspa except in Exps. 4 and 5 where PSbMV-resistant cv. Yarrum was also used (as a control treatment). Numbers of replicates per treatment is five (Exps. 1 and 2), four (Exp. 3) or three (Exps. 4 and 5). Mulch treatment was added straw mulch (2 t/ha). Insecticide treatment consisted of combined foliar applications of α-cypermethrin (Fastac at 500 mL/ha) and imidacloprid (Confidor at 170 mL/ha). + = Treatment applied, - = Treatment not applied, ns = not statistically significant. Final % PSbMV incidence and seed yield values followed by the same letter (a–d) are not significantly different at *p* < 0.05. Final % PSbMV incidence values statistically analysed based on angular transformed percentages (shown in [6]).

## Data Availability

Not applicable for reviews based entirely upon previously published information.
